# Early and late phases of liver sinusoidal endothelial cell (LSEC) defenestration in mouse model of systemic inflammation

**DOI:** 10.1186/s11658-024-00655-w

**Published:** 2024-11-11

**Authors:** Izabela Czyzynska-Cichon, Jerzy Kotlinowski, Oliwia Blacharczyk, Magdalena Giergiel, Konrad Szymanowski, Sara Metwally, Kamila Wojnar-Lason, Ewelina Dobosz, Joanna Koziel, Malgorzata Lekka, Stefan Chlopicki, Bartlomiej Zapotoczny

**Affiliations:** 1 Jagiellonian Centre for Experimental Therapeutics (JCET), Jagiellonian University, Bobrzynskiego 14, 30-348 Krakow, Poland; 2 Department of General Biochemistry, Faculty of Biochemistry, Biophysics and Biotechnology, Jagiellonian University, Gronostajowa 7, 30-387 Krakow, Poland; 3 Institute of Nuclear Physics Polish Academy of Sciences, 31342 Krakow, Poland; 4 Centre for Nanometer-Scale Science and Advanced Materials (NANOSAM), Faculty of Physics, Astronomy, and Applied Computer Science, Jagiellonian University, Krakow, Poland; 5 Department of Microbiology, Faculty of Biochemistry, Biophysics and Biotechnology, Jagiellonian University, Gronostajowa 7, 30-387 Krakow, Poland; 6 Department of Pharmacology, Jagiellonian University Medical College, Grzegorzecka 16, 31-531 Krakow, Poland

**Keywords:** Mcpip1, Fenestration, Actin cytoskeleton, Liver, LSEC, Forced sieving

## Abstract

**Background:**

Liver sinusoidal endothelial cells (LSECs) have transcellular pores, called fenestrations, participating in the bidirectional transport between the vascular system and liver parenchyma. Fenestrated LSECs indicate a healthy phenotype of liver while loss of fenestrations (defenestration) in LSECs is associated with liver pathologies.

**Methods:**

We introduce a unique model of systemic inflammation triggered by the deletion of Mcpip1 in myeloid leukocytes (Mcpip1^fl/fl^LysM^Cre^) characterised by progressive alterations in LSEC phenotype. We implement multiparametric characterisation of LSECs by using novel real-time atomic force microscopy supported with scanning electron microscopy and quantitative fluorescence microscopy. In addition, we provide genetic profiling, searching for characteristic genes encoding proteins that might be connected with the structure of fenestrations.

**Results:**

We demonstrate that LSECs in Mcpip1^fl/fl^LysM^Cre^ display two phases of defenestration: the early phase, with modest defenestration that was fully reversible using cytochalasin B and the late phase, with severe defenestration that is mostly irreversible. By thorough analysis of LSEC porosity, elastic modulus and actin abundance in Mcpip1^fl/fl^LysM^Cre^ and in response to cytochalasin B, we demonstrate that proteins other than actin must be additionally responsible for inducing open fenestrations. We highlight several genes that were severely affected in the late but not in the early phase of LSEC defenestration shedding a light on complex structure of individual fenestrations.

**Conclusions:**

The presented model of LSEC derived from Mcpip1^fl/fl^LysM^Cre^ provides a valuable reference for developing novel strategies for LSEC refenestration in the early and late phases of liver pathology.

**Supplementary Information:**

The online version contains supplementary material available at 10.1186/s11658-024-00655-w.

## Introduction

The liver, owing to its morphology and specific double blood supply, serves as the body’s filtration system, metabolic and detoxification centre, nutrient store and homeostasis regulator. A particular role in liver function is played by liver sinusoidal endothelial cells (LSECs), which line hepatic capillaries. As the unique front line between blood and liver parenchyma, LSECs are constantly exposed to dietary molecules, metabolites and antigens. The defining feature of LSECs, distinguishing them from endothelial cells in other vascular beds, is their transcellular pores, called fenestrations (or Latin ‘fenestrae’). These nanostructures – 50–350 nm in diameter – participate in the passive, size-regulated and bidirectional transport between the microcirculation and the liver parenchyma, determining healthy liver phenotype [1–3]. In particular, the physiological porosity of LSECs provides hepatocytes with access to circulating chylomicron remnants, thereby maintaining lipid homeostasis while LSEC defenestration may induce hypertriglyceridemia [4, 5]. Indeed, plasmalemma vesicle-associated protein (PLVAP)-deficient mice, displaying severe defenestration of LSECs, also display severe hyperlipoproteinemia [6], suggesting that LSECs fenestration represent a previously not appreciated target for the regulation of undisturbed passage of triglyceride-rich lipoproteins [7].

The partial loss of LSEC fenestrations (defenestration), along with various phenotypic changes of LSECs, is considered a hallmark of LSEC dysfunction. It accompanies the development of sinusoidal capillarisation during various liver pathologies, including liver cirrhosis, fibrosis, hepatosteatosis and hepatitis [2, 8]. Moreover, reduced porosity of LSECs has been observed in non-liver diseases, such as diabetes [9], cardiovascular diseases, including atherosclerosis or heart failure [5, 10], as well as in LPS-induced inflammation [11, 12]. This reduction in porosity contributes to liver pathology in these conditions as well as to hyperlipidemia. Finally, LSEC defenestration is a hallmark of ageing in the liver and is accompanied by the thickening of the endothelium and basement membrane formation in the process of pseudocapillarisation [13, 14].

Given the important role of LSEC fenestration in the maintenance of hepatic sinusoid homeostasis, several studies suggested that restoration of LSEC porosity may prove useful as a therapeutic target to treat various liver pathologies [15], to improve liver regeneration [16], to limit ageing-dependent liver disease [17]. At 50 years since the first depiction of fenestrations in LSECs [18], the mechanisms regulating maintenance of fenestrations are now better but still not fully understood [1, 2, 19–22]. In particular, it is not clear to what extent LSEC defenestration is reversible by pharmacological tools. Numerous reports have shown that depolymerisation of the actin cytoskeleton induced in vitro by actin-disrupting agents, such as cytochalasin B, latrunculin or swinholide, forces LSECs to open new fenestrations (even up to 300% in number) (Tables in [1, 2, 21]). The treatment was even successful in the refenestration of LSECs which were defenestrated as a result of long-term culture [22]. Furthermore, several compounds were shown to be effective in the refenestration of pseudocapillarized LSECs, e.g. sildenafil, nicotinamide mononucleotide or serotonin [2, 16, 23, 24]. However, refenestration strategies using cytochalasin B were reported to not be completely successful in LSEC defenestration associated with chronic heart failure [10]. Moreover, the inhibition of calmodulin-dependent myosin phosphorylation using ML-7 dihydrochloride [20] or tubulin stabilisation using Taxol [25] abolished the refenestration effect of cytochalasin B. Different patterns of LSEC response to cytochalasin B in various pathologies associated with LSECs defenestration indicate that the effectiveness of actin-targeted therapy to restore fenestrations in LSEC may depend on the stage of LSEC phenotypic changes. Up until now, there is no good genetic model to study defenestration in vitro and to test the refenestration strategies.

We took advantage of a well-characterised model of conditional deletion of Monocyte chemoattractant protein-1 induced protein 1 (Mcpip1) in murine myeloid cells (Mcpip1^fl/fl^ LysM^Cre^) mice model. Mcpip1 is an RNase involved in the control of inflammatory processes via direct degradation of mRNA molecules coding for proinflammatory cytokines, such as Il-1b and Il-6 [26, 27]. Mcpip1 deficient mice spontaneously develop systemic inflammatory responses leading to splenomegaly, lymphadenopathy, hyperimmunoglobulinemia and ultimately death within 3 months [28]. In contrast to total Mcpip1 knockout mice, Mcpip1 LysM^Cre^ animals characterised by the deletion of Mcpip1 in myeloid leukocytes were used here. The advantages of Mcpip1^fl/fl^ LysM^Cre^ mice strain are related to low-grade proinflammatory phenotype manifested in 3-month-old mice that gradually and spontaneously develop into fully blown inflammatory syndrome but at a late age compared with global Mcpip1 knockout mice [29]. Mice with deletion of Mcpip1 in myeloid leukocytes were also characterised by B lymphocytes and plasma cells expansion, spontaneous production of autoantibodies, leading to skin inflammation, pneumonitis and lupus-like syndrome [30]. More recently, in the same mice strain Szukala et al., demonstrated spontaneous development of skin allergic reactions, strongly influenced by gut dysbiosis and enhanced systemic dissemination of bacteria [31]. In the skin of Mcpip1 LysMCre mice, researchers detected increased infiltration of Th2 lymphocytes, eosinophil and mast cells. Recently, we showed that the deletion of Mcpip1 in myeloid leukocytes is significant in inducing severe changes in the livers of 6-month-old Mcpip1^fl/fl^ LysM^Cre^ mice, including impaired lipid transport, glucose metabolism, profound liver fibrosis and lower level of fatty acids oxidation [32].

The aim of the present work was to better understand the pathophysiology of the progression of LSEC defenestration in response to systemic inflammation with the aim to validate the hypothesis of the distinct phenotype of LSECs in the early to late phases of liver pathology development. For that purpose, we took advantage of the well-characterised Mcpip1^fl/fl^ LysM^Cre^ model that displays multiple manifestations of a systemic inflammatory response [30, 31, 33, 34], allergic inflammation [31] as well as impaired lipid homeostasis and liver fibrosis [32]. These pathologies indicate a possible alteration in LSEC porosity, that has never been studied in this model. A detailed analysis of LSEC fenestration using a novel imaging method based on atomic force microscopy (AFM) as compared with a well-established method using scanning electron microscopy (SEM) supplemented with quantitative fluorescence microscopy of actin filaments, allowed performing a detailed characterisation of LSECs in Mcpip1^fl/fl^ LysM^Cre^ mice model. We provided a discussion of biomechanics, ‘forced sieving’ response, and transcriptomic changes in LSECs, all to characterise early and late phases of defenestration in Mcpip1^fl/fl^ LysM^Cre^ model. Finally, phases of LSEC defenestration were discriminated on the functional levels by full and partial recovery in response to cytochalasin B.

## Materials and methods

### Animals, diets, and genotyping

Mcpip1^fl/fl^ LysM^Cre^ mice were obtained by crossing floxed MCPIP-1 mice with LysMCre mice as described previously [32] and bred in the Faculty of Biochemistry, Biophysics and Biotechnology at the Jagiellonian University in Kraków, Poland. Animals were housed in a temperature-controlled environment (22 ± 2 °C) with a 12 h light/dark cycle and fed ad libitum. All experiments were performed on Mcpip1^fl/fl^ LysM^Cre^ and age‐matched control male mice (Mcpip1^fl/fl^) at the ages of 3 and 6 months, representing early- and late stages of liver pathology, respectively [29]. The experiments were performed to achieve at least three repetitions per group. All procedures involving animals were conducted according to the Guide for the Care and Use of Laboratory Animals (Directive 2010/63/EU of the European Parliament).

### Cell isolation and culture

LSECs were isolated according to the procedure described in detail elsewhere [35, 36]. Briefly, mice were euthanized by intraperitoneal injection of ketamine (100 mg/kg) and xylazine (10 mg/kg) mixture. The portal vein was cannulated and the blood was flushed out through the incision in the inferior vena cava with the perfusion buffer (142 mM of NaCl, 6.708 mM of KCl, 9.6 mM of HEPES and 6 mM of NaOH) at 37 °C. Next, the digestion buffer containing 0.02 mg/mL of Liberase (Roche, Switzerland) was applied to obtain suspension of liver cells. The LSEC population was purified by a series of centrifugations followed by immunoseparation using LSEC-specific CD146 magnetic MicroBeads (MACS, MiltenyiBiotec, Germany). After isolation, cells were seeded at the desired density and incubated overnight at 37°C in endothelial cell growth medium (EGM)-2 cell culture medium (Lonza, Basel, Switzerland), unless otherwise stated. Isolated and purified cells were seeded on round 13 mm glass coverslips (35 000 cells/slide) or Ø 35 mm Petri dishes (70 000 cells/slide) and cultured overnight (12–14 h). The experiments on living cells were started after 5 h and were finished no later than 20 h after plating, as introduced before [37]. We did not observe variances in the cell responsiveness to drugs in the selected time frame.

### Atomic force microscopy

AFM measurements were conducted in three different modalities: (A) quantitative imaging (QI) mode imaging of fixed cells, (B) QI mode imaging of living cells and (C) force spectroscopy, described in detail in Supplementary Information. Briefly, the culture medium was replaced with the fresh one (EGM-2, Lonza) with the addition of 25 mM of HEPES buffer (4-(2-Hydroxyethyl)piperazine-1-ethane-sulfonic acid, Sigma-Aldrich) to prevent changes in pH of culture medium during the measurements (ambient atmosphere). To test the responsiveness of LSECs to drug treatment, the compound was injected into the cell culture and imaging of the same area was continued. The set of images was presented in the form of supplementary videos. The force spectroscopy mode of AFM was used to determine the elastic modulus which is apparent Young’s modulus, as not all requirements of the contact mechanics can be fulfilled for biological objects [38]. Force–distance curves were acquired in the preselected central area of the cell with sharp and hemispherical probes in force-volume mode [39]. The final values of elastic modulus were expressed as a mean and standard deviation from all measured cells in the group.

### Fluorescence microscopy

Cells were cultured on fibronectin coated Ø13 mm glass coverslips in EGM-2 (Lonza) medium for 12–14 h. Then, the medium was replaced with the medium with or without the drug and after 30 min samples were fixed using 3.6% paraformaldehyde for 30 min. The fixative was rinsed with phosphate buffered saline (PBS) and stored for up to 14 days. Before imaging, cells were permeabilised using 0.1% Triton X-100 for 4 min and rinsed again with PBS. Actin was labelled using phalloidin-AlexaFluor488 (Invitrogen) in PBS (1:200) for 60 min. Cell nuclei were labelled using Hoechst 34580 (Invitrogen) (0.2 μg/ml) in PBS buffer for 10 min. Cells were rinsed four times with PBS for 5 min. Finally, samples were mounted on glass slides using Prolong Diamond Antifade Mountant (Thermo Fisher Scientific). Epifluorescence images were acquired using an oil immersion × 100 objective (1.4 NA) using Olympus IX53 fluorescence microscope.

The level of actin polymerisation was quantified using FilamentSensor2.0 free software [40]. 10–15 images were collected in different areas of a sample using acquisition time of 100–150 ms. From acquired images representative cells were cut using Fiji software [41]. Fibres’ abundance per cell was calculated and presented as the number of detected fibres and their length. The minimal length of the fibre was arbitrarily set to 50 px (~ 3.5 µm) to cut off the shortest fibres that dominated the resulting data and were considered as noise and to focus on long fibres instead. Fibres detected as cell edges were excluded from the analysis. No additional image filtering was applied in the software.

### Scanning electron microscopy (SEM)

Cells were cultured on Ø13 mm glass coverslips in EGM-2 (Lonza) medium for 12–14 h. The medium was changed to the medium with the drug and after the treatment samples were fixed using 2.5% glutaraldehyde (GA) in distilled water (dH_2_O) and stored in GA at 4 °C. Samples from the same isolation, obtained always in pairs of one control and one Mcpip1 KO were processed on the same day. Each pair of bio-replications was processed individually. Prior to SEM imaging, samples were rinsed with dH_2_O to remove GA. Then, cells were stained with a 1% OsO_4_ solution in H_2_O (Merck, Germany) for 1 h, and rinsed three times in dH_2_O afterwards. Samples were dehydrated in 50%, 70%, 96% and 100% ethanol solutions (analytical standard, POCH, Poland) and finally with hexamethyldisilazane (Merck, Germany) overnight. Next, samples were coated with an approximately 5 nm gold layer using a rotary-pumped sputter coating (Q150RS, Quorum Technologies, UK). Cells were studied using SEM (Merlin Gemini II, Zeiss, Germany) at a current of 90 pA and voltage of 5 kV, using a HE-SE detector. We did not analyse fenestration diameters owing to randomly occurring sample preparation artefacts, such as cracks in the perinuclear zone, poor contrast or observed elongation of part of fenestrations. Still, we could easily distinguish between fenestrations and the image artefacts, therefore, we provided information on fenestration frequency. Analysis of fenestration frequency, i.e. the number of fenestrae per area of cell was calculated using the manual method described before [42]. Briefly, the area of cells was calculated in Fiji free software and a number of fenestrae was manually counted using the CellCounter plugin to Fiji.

### Next generation sequencing NGS

For analysis of LSEC transcriptome, datasets published by Zurawek et al., were used [29]. Functional annotation of differentially expressed genes (≥ 1.5-fold change) was conducted using DAVID tools version v2023q4 [43]. Gene lists were searched using official gene symbol annotation. A background dataset of *Mus musculus* was applied for analyses. DAVID tool mapped up- and downregulated genes to Biological Process (BP) Gene Ontology (GO) terms. The heat map shows the *z*-score calculated for differentially expressed genes related to LSEC function, structure and development of fenestrae. Functional enrichment analysis was performed using Cytoscape software version 3.10.1.

### Reverse transcription-quantitative polymerase chain reaction (RT-qPCR)

Total RNA from LSEC was isolated using Fenozol (A&A Biotechnology). For reverse transcription, 500 ng of RNA, oligo(dT) primers (Promega) and M-MLV reverse transcriptase (Promega) were used. Quantitative PCR (qPCR) was carried out using SYBR Green Master Mix (Sigma-Aldrich) and a QuantStudio qPCR System (Applied Biosystems). Gene expression was normalized to Ef2, and then, the relative transcript level was quantified by the 2^ΔCt^ method. Primer sequences (Genomed/Sigma) are listed in Supplementary Table 1.

### Statistical analysis

The statistical significance was calculated with Student’s *t*-test for unpaired data. AFM: the box charts show the mean (line) and range central 50% of data points, the whiskers indicate 5% and 95% data range, respectively. qPCR: the bar charts show the average and the whiskers standard deviation. *p* Value less than 0.05 was considered significant. * p < 0.05; ** p < 0.01, *** p < 0.001.

## Results

### Progression of LSEC defenestration in Mcpip1 KO mice

**Fig. 1:**
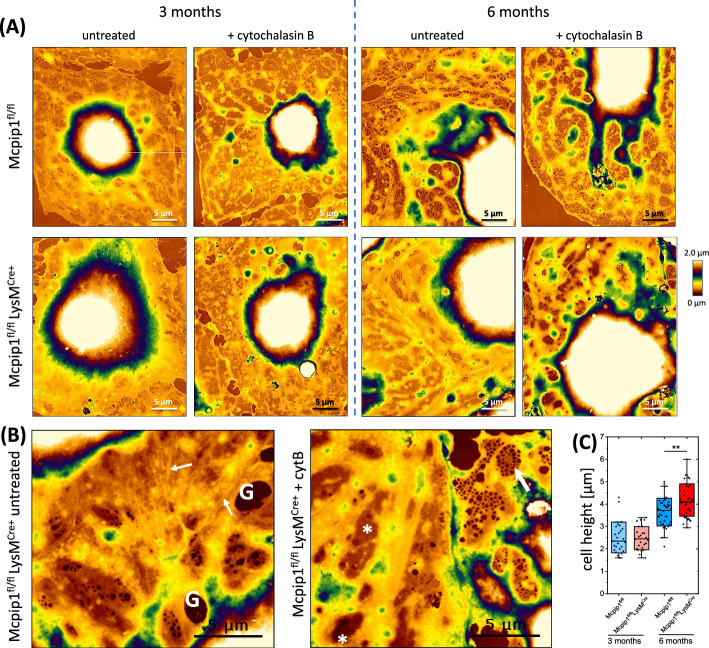
Topography images of representative LSECs isolated from 3-month-old and 6-month-old Mcpip1 KO and control mice were measured using QI AFM. **A** The first row represents LSEC isolated from control, Mcpip^fl/fl^ mice; the second row from Mcpip1 KO, Mcpip1^fl/fl^ LysM^Cre^ mice. Additionally, the effect of 30 min treatment with 21 µM cytochalasin B was presented, showing weaker responsiveness, i.e. induction of fewer fenestrae in the Mcpip1 KO group. Images are in scale for comparison. 30 × 35 µm^2^, 350 × 408 pixels. **B** High-magnification images of untreated and cytochalasin B-treated LSECs from the Mcpip1 KO group. Sieve plates without opened fenestrations were indicated using asterisks, small arrows show examples of cytoskeletal fibres that were visible only in the untreated group. A large arrow indicates a sieve plate filled with fenestrations. ‘G’ indicates a gap, a large hole that was not classified as functional fenestration. Images are in scale for comparison. 18 × 16 µm.^2^, 204 × 184 pixels (bicubic interpolation). **C** Cell height of control and Mcpip1 KO LSECs in both time points (*n* = 3 animals). ** *p* < 0.01

The AFM-based methodology was applied to characterise the morphology of fixed LSECs isolated from 3-month-old and 6-month-old Mcpip1^fl/fl^ (control) and Mcpip1^fl/fl^ LysM^Cre^ (Mcpip1 KO) mice. LSECs in all groups exhibited characteristic cobblestone morphology with bulging nuclei and thin (< 400 nm) peripheries (Fig. 1A). Outside of the nuclear region LSECs were perforated with fenestrations gathered in sieve plates. LSECs in the Mcpip1 KO group have fewer fenestrations, but sieve plates were still observed. High-magnification images identified abundantly present cytoskeletal fibres in the 6-month-old Mcpip1 KO group that vanished after the cytochalasin B treatment (Fig. 1B). Additionally, areas resembling sieve plates, but without opened fenestrations within were identified after the cytochalasin B treatment (Fig. 1B, asterisk). The cell central area containing the cell nucleus was larger in the Mcpip1 KO group of both time points, although the maximal height varied only in older animals. LSECs isolated from 6-month-old Mcpip1 KO mice had a mean cell height of 4.2 ± 0.75 µm as compared with 3.6 ± 0.54 µm in LSECs from control mice (*n* = 38 cells/group) (Fig. 1C).

**Fig. 2:**
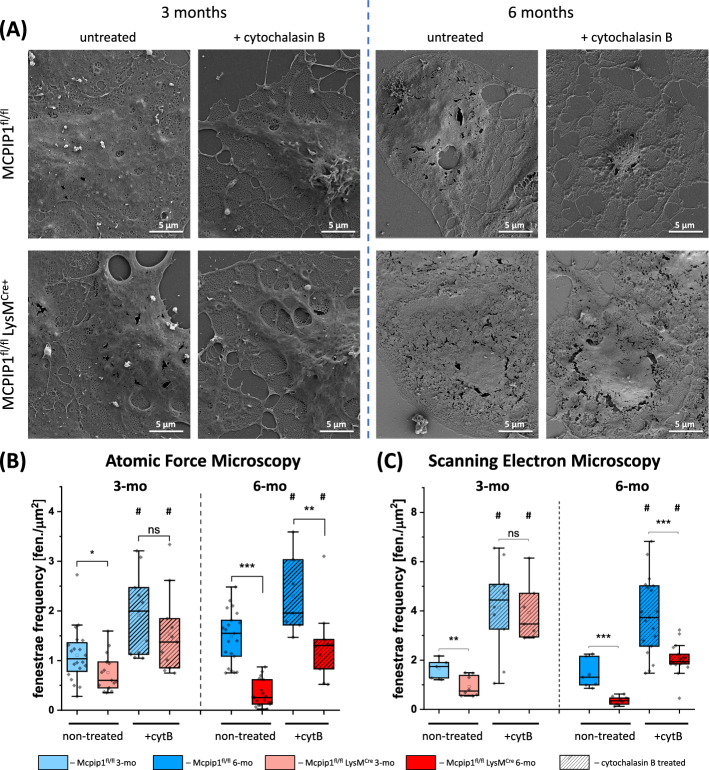
Comparison of LSEC porosity measurements in Mcpip1 KO and control mice using AFM and SEM. **A** Representative scanning electron microscopy images of LSECs isolated from 3-month-old and 6-month-old mice. **B,C** The porosity of LSECs expressed as a fenestrae frequency, i.e. the number of fenestrations per area of cell, as measured using **(B)** atomic force microscopy (AFM) and **(C)** scanning electron microscopy (SEM) for GA-fixed cells. Additionally, the effect of 30 min treatment with 21 µM cytochalasin B was presented, showing weaker responsiveness, i.e. induction of less fenestrae in the Mcpip1 KO group. Each point represents a single cell. The box charts show mean ± 25–75% of data points and the whiskers 5% and 95%. * *p* < 0.05; ** *p* < 0.01, *** *p* < 0.001. Additionally, # indicates significance of *p* < 0.01 after cytochalasin B treatment versus respective non-treated genotype. *n* = 3–6 animals

The porosity of LSECs (expressed as a fenestrae frequency) isolated from Mcpip1 KO mice was decreased compared with age-matched control mice, as evidenced by AFM and SEM (Fig. 2). In LSECs isolated from 3-month-old control mice, the observed mean fenestrae frequency of 1.11 ± 0.55 fen./µm^2^ was typical for primary murine LSECs measured using AFM [44]. A larger, but still physiological frequency value of 1.41 ± 0.45 fen./µm^2^ was detected in LSECs isolated from 6-month-old control animals (Fig. 2A). Relative to the controls, in Mcpip1 KO the number of LSEC fenestrations was lower in both 3-month-old (0.75 ± 0.37 fen./µm^2^) and 6-month-old mice (0.33 ± 0.26 fen./µm^2^). The results obtained from AFM were compared with those obtained from a well-established SEM-based analysis for dehydrated samples (Fig. 2A,C) and resulted in similar trends. The analysis of SEM images demonstrated a clear 50% reduction in fenestrae frequency in the 3-month-old Mcpip1 KO group (0.83 ± 0.42 fen./µm^2^), compared with the control group (1.66 ± 0.41 fen./µm^2^). LSECs in the 6-month-old Mcpip1 KO group displayed more severe defenestration, characterised by approx. 80% reduction in the number of fenestrations (SEM, control: 1.42 ± 0.56 fen./µm^2^); SEM, Mcpip1 KO: 0.35 ± 0.16 fen./µm^2^).

### Full and partial recovery of LSECs’ fenestrations in 3-month-old and 6-month-old control and Mcpip1 KO mice in response to cytochalasin B

Fenestrations are dynamic structures that are continuously formed, moved within the cell to be finally fused within minutes to hours [45]. This dynamics and the ability to form new pores constitute an important feature of LSEC functionality. The gold standard to test this ability by disclosing cell potential to form fenestrations is cytochalasin B, which in 15–30 min causes actin disruption, allowing to induce up to 300% of fenestrations [2, 46, 47]. Here, the treatment with cytochalasin B resulted in a two-fold increase in the mean fenestrae frequency in both the 3-month-old and 6-month-old control groups **(**Fig. 2**)**. Defenestrated LSECs, isolated from 3-month-old Mcpip1 KO, responded normally to cytochalasin B, by inducing numerous fenestrations, (1.53 ± 0.81 fen./µm^2^) reaching the values of treated control cells (1.91 ± 0.81 fen./µm^2^). In contrast, for 6-month-old Mcpip1 KO treated with cytochalasin B, the value of 1.32 ± 0.74 fen./µm^2^ indicated only partial recovery compared to treated control, where almost twice as many fenestrations were observed (2.30 ± 0.78 fen./µm^2^). The maximal observed values of fenestration frequency were in the range of the untreated control group and far below values for cytochalasin B treated control **(**Fig. 2A**)**. These results indicate that in LSECs isolated from 6-month-old KO mice not only the basal level of porosity was reduced, but also the LSEC potential to induce new fenestrations in response to cytochalasin B was impaired. Notably, data obtained with the SEM technique reflected the same trends as AFM-based analysis (Fig. 2B).

AFM remains the exclusive technique allowing us to track the changes in morphology in living LSECs, including the real-time visualisation of drug response. To assess the dynamics of new fenestration formation in response to cytochalasin B over time, living LSECs isolated from Mcpip1 KO mice were analysed using AFM (Supplementary videos 1–4 and Supplementary Fig. 1).

Firstly, a region of interest (ROI) that covered representative LSEC was selected. The initial number of fenestrations in ROI reflected observations in fixed cells and was lower for 6-month-old Mcpip1 KO. However, the fenestration dynamics was preserved, as new fenestrations were continuously formed and disappeared. Cytochalasin B induced rapid changes in cellular morphology with the depolymerisation of thick fibres observed within the first 10 min (Supplementary Fig. 1, arrowheads). The effect of cytochalasin B on a number of fenestrations reached the maximum after 15–30 min as reported previously [46, 48]. Exposed to Cytochalasin B, LSECs isolated from 3-month-old Mcpip1 KO mice formed numerous fenestrations and fenestrae-forming centres [49] (Supplementary Fig. 1, arrows). The number of newly formed fenestrations was much smaller in the 6-month-old Mcpip1 KO group (Supplementary Fig. 1, Supplementary videos 3,4) when compared with the 3-month-old Mcpip1 KO group. Even an incubation time longer than 30 min with cytochalasin B did not increase the number of fenestrations. The cell height outside the nuclear region was reduced and shapes resembling sieve plates were identified (Supplementary Fig. 1, asterisk), but the membrane within was often sealed without opening new fenestrations.

### 3.3. Relationship between nanomechanical properties of LSECs, microarchitecture of the cytoskeleton and fenestrations in response to cytochalasin B

To verify whether there is a link between the mechanical properties of LSECs in the progress of defenestration and altered response to cytochalasin B, the cell stiffening (an increase of elastic modulus), the level of actin polymerisation (abundance of actin fibres) and the forced sieving properties (load force dependent deformability of fenestrations) were investigated.

#### 3.3.1. Comparable effects of cytochalasin B on LSEC stiffness in 3- and 6-month-old control and Mcpip1 KO mice

Stiffening of the liver is accompanied by LSEC defenestration [50]. Moreover, it was recently reported that LSECs' porosity depends on the mechanical properties of their environment [51]. Here, the elasticity of LSECs was studied in vitro using pyramidal and hemispherical indenters, allowing deep and hollow indentations. The deep indentation of more than 1000 nm results in the convoluted signal from the cell membrane, cortical layer and the cell nucleus (sometimes also the effect of the stiff substrate) [52–54]. To collect the information from the cell cortex only and to focus on actin input to elastic modulus, parallel experiments using hemispherical probes with large tip radius (R = 5 µm) were performed.

**Fig. 3:**
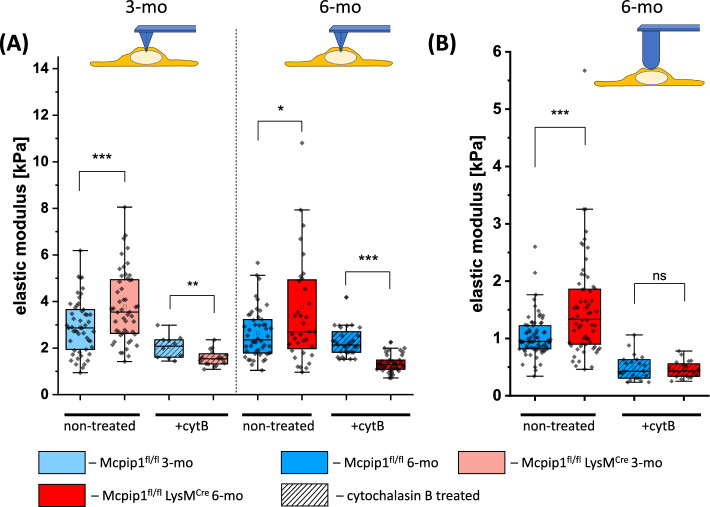
Elastic modulus of LSEC isolated from control and Mcpip1 KO mice. Nanomechanical properties of LSEC quantified by the elastic modulus calculated for (**A**) the conical indenter (F = 1.0 nM) and (**B**) the hemispherical indenter (r = 5.0 µm, F = 2.0 nN). Two time points of 3-month-old and 6-month-old mice were compared. Additionally, the effect of 30 min of cytochalasin B was determined. *n* = 3–6 animals

The Young’s modulus of control cells, measured using a cantilever with a conical tip, did not differ between cells from 3-month-old and 6-month-old mice and presented values of 2.9 ± 1.1 kPa and 2.6 ± 1.0 kPa, respectively (Fig. 3A). The stiffening of LSECs in the Mcpip1 KO group was observed, however, this alteration was not progressive over time. The mean values of 3.8 ± 1.5 kPa (3-month-old Mcpip1 KO) and 3.8 ± 2.9 kPa (6-month-old Mcpip1 KO) were significantly larger than the respective controls. After depolymerisation of actin with cytochalasin B, elastic modulus reduction and a narrowing of the data distribution in all studied groups were observed (Fig. 3A). Interestingly, initially stiffer LSECs isolated from Mcpip1 KO became softer than the age-matched controls after treatment with cytochalasin B, reaching 2.3 ± 0.7 kPa versus 1.3 ± 0.4 kPa, for 6-month-old control and 6-month-old Mcpip1 KO groups, respectively. The observed difference might be connected with the softening of cell nuclei in the Mcpip1 KO group, as the treatment with cytochalasin B removes the actin input to the cell stiffness. Similar to our observations, the softening of the nuclear envelope was reported before as a response to mechanical stress induced by increased actin polymerisation [55, 56].

The measurements using hemispherical probes confirmed stiffening of the cell cortex based on a significantly increased value of elastic modulus for LSECs isolated from 6-month-old Mcpip1 KO mice compared to controls (Fig. 3B). Additionally, treatment with cytochalasin B reduced elastic modulus in both control and Mcpip1 KO groups to a comparable level, reflecting similar levels of actin depolymerisation in the cortical layer (Fig. 3B). This result may suggest that the reversibility of LSEC defenestration which was distinct in 3-month-old and 6-month-old Mcpip1 KO mice is not directly related to cell stiffness; here the elasticity was effectively reduced in both 3-month-old and 6-month-old Mcpip1 KO mice to a similar level.

#### 3.3.2. Different actin cytoskeleton microarchitecture of 6-month-old control and Mcpip1 KO mice

**Fig. 4:**
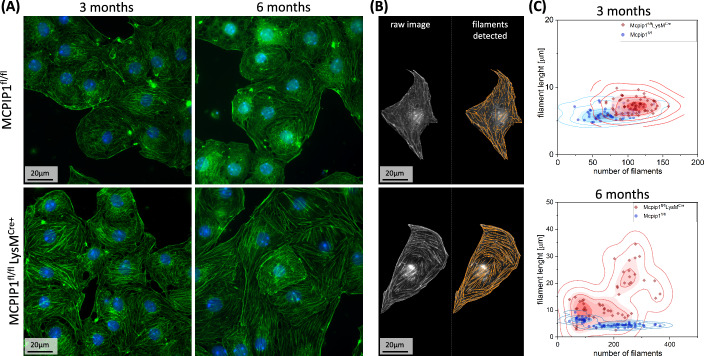
Quantitative fluorescence of actin filaments allows distinguishing between Mcpip1^fl/fl^ (top) and Mcpip1^fl/fl^ LysM^Cre^ (bottom). **A** The panel presents representative images of LSECs from 3-month-old and 6-month-old Mcpip1^fl/fl^ and Mcpip1^fl/fl^ LysM^Cre^ mice. **B** A single cell was extracted from the image and analysed using FilamentSensor2.0 software. **C** The results of filaments length and number of over 50 cells per group are presented as Kernel density diagrams (blue—Mcpip1^fl/fl^, red—Mcpip1^fl/fl^ LysM^Cre^). *n* = 3 animals

It was reported before that increased elastic modulus is accompanied by a higher level of actin polymerisation and the presence of longer actin fibres [54, 57, 58]. Our findings agree with those reports showing a much higher abundance of long actin fibres in Mcpip1 KO group (Fig. 4). It is noteworthy that being primary cells, LSECs show high heterogeneity even in control groups and individual LSECs had long actin fibres (Fig. 4A). Nevertheless, a comparison of over 50 individual cells (*n* = 3 of biological repetitions) indicated that there was a significant increase in the length and number of the actin fibres in Mcpip1 KO mice at both time points (Fig. 4B,C). The average number and length of the detected fibres: for 3-month-old mice were 86.6 ± 11.1, 5.2 ± 1.7 µm (control) and 104.1 ± 14.0, 7.6 ± 1.7 µm (KO); for 6-month-old animals were 72.2 ± 17.7, 12.5 ± 6.0 µm (control) and 82.2 ± 14.2, 13.2 ± 7.7 µm (KO). Despite the mean values of filament length being similar in the 6-month-old controls and Mcpip1 KO, the 2D Kernel density graphs revealed significant heterogeneity within the KO group. Notably, a distinct cell subpopulation characterised by mean fibre lengths of 22 ± 3.2 µm was identified (Fig. 4C). These results indicate that elastic modulus values did not allow us to distinguish between the early and late defenestration phases, but the microarchitecture of the actin cortex shows significant changes in the late stage of LSEC pathology compared to control mice and 3-month-old Mcpip1 KO mice.

In addition to untreated cells, the effect of cytochalasin B on actin filaments was investigated. The significant depolymerisation of long actin filaments after 30 min of treatment was observed in all groups and only a few thick fibres remained (Supplementary Fig. 2). The trend towards preserving a higher level of actin polymerisation in cytochalasin B treated Mcpip1 KO in comparison to respective controls was preserved; however, in both groups, only rudimentary and short actin filaments with lengths below 5 µm were observed after the treatment. These results indicate that the depolymerisation of actin using cytochalasin B has similar effects on actin distribution, organisation and mechanical properties of LSECs from 3-month-old and 6-month-old Mcpip1 KO, but the potential to induce new fenestrations is impaired only in 6-month-old Mcpip1 KO. Therefore, the observed discrepancies must involve mechanisms other than directly related to actin polymerisation.

#### 3.3.3 Impaired *forced sieving* in LSECs from 6-month-old control and Mcpip1 KO mice

**Fig. 5:**
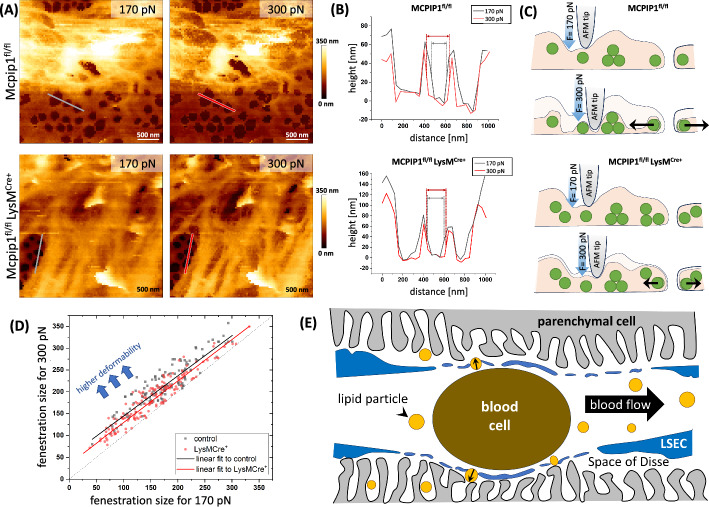
Low deformability of LSECs from Mcpip1^fl/fl^ LysM^Cre^ mice indicates altered forced sieving. **A** Representative images of individual sieve plates measured in living LSEC using QI AFM. The images are reconstructed for load force of 170 nN and 300 pN which represents ~ 50% and ~ 80% of the maximum load force, respectively. **B** Selected cross-section indicated in (**A**) was presented showing the differences between the load force used. The enlargement of fenestrations was greater in the control rather than the Mcpip1 KO group, which was schematically illustrated in (**C**). **D** shows the distribution of > 200 fenestrations compared in a one-to-one manner for selected load force values with a linear fit. **E** The scheme illustrating the forced sieving of lipoproteins through fenestrations is a modification of a scheme shown in [59]. *n* = 3 animals

The intrahepatic blood flow dynamics in sinusoids engage two processes: ‘forced sieving’ and ‘endothelial massage’ [7, 59, 60]. Their concept was made on the basis of the anatomical ground of sinusoids and blood cell size and illustrated (Fig. 5E). It is thought that blood cells are passing in a row exerting perpendicular force acting on the sinusoidal wall [61]. When the cell approaches, the sinusoidal wall contracts and the fluids and particulate material are removed from the space of Disse to the bloodstream [62]. When the cell is passing, the pressure is released and the sinusoidal wall relaxes to its original morphology. As a result, the fluid and colloidal particles are sucked into the space of Disse and exposed to the hepatocytes’ microvilli [61]. We hypothesise that not only the number and diameter of fenestrations play a role in the transport through fenestrations, but also their deformability in response to the force exerted by the flowing blood. To address the ‘forced sieving’ and ‘endothelial massage’ concepts, QI AFM imaging of living LSECs was implemented to assess fenestration deformability. The imaging of multiple sieve plates of LSECs isolated from 6-month-old control and Mcpip1 KO mice was performed using load forces of 170 pN and 300 pN (Fig. 5). The lower value ensures clear imaging of fenestrations with minimal noise, while the larger load force is aligned with typical AFM measurement conditions [48, 63]. Such an approach guaranteed that fenestrations remained distinct in the resulting image, avoiding any appearance of merging.

Individual fenestrations changed their diameters with increasing pressure. The level of these changes was greater in the control group than in the Mcpip1 KO group. It indicated lower deformability of fenestrations in Mcpip1 KO model. The direct comparison of over 200 fenestrations per group (*n* = 3 animals) allowed for quantifying the observed discrepancies in the deformability between the groups (Fig. 5A–C). The linear fit of the function *y* = *ax* + *b* to the data of fenestration diameter measured for 170 pN versus 300 pN describes the level of deformability. Parameter *a* reflects the slope of the curve and indicates the size-dependent changes in deformability. Here, *a* was similar in both groups and equalled 0.918 and 0.925 for the control and KO, respectively. The parallel character of both lines and values of *a* parameter close to 1 suggests that irrespective of the initial fenestration size, the enlargement rate is similar in both groups. The parameter *b* reflects the level of fenestration enlargement resulting from an increase in the load force from 170 to 300 pN. The large, over 35% lower values of parameter *b* in the Mcpip1 KO group highlight the lower deformability of fenestrations in this group (b = 42.0 ± 4.1 in the control versus b = 57.3 ± 6.6 in the Mcpip1 KO group). Interestingly, when actin filaments were depolymerised and elastic modulus reduced using cytochalasin B, the difference in deformability of fenestrations between the Mcpip1 KO and the control groups vanished (Supplementary Fig. 3). In summary, the increased elastic modulus of LSECs in the Mcpip1 KO group (Fig. 3) pairs with the less deformable surface of the cell peripheries, including less deformable fenestrations.

### 3.4 Progressive alterations in LSEC genes in 3- and 6-month-old Mcpip1 KO mice

**Fig. 6:**
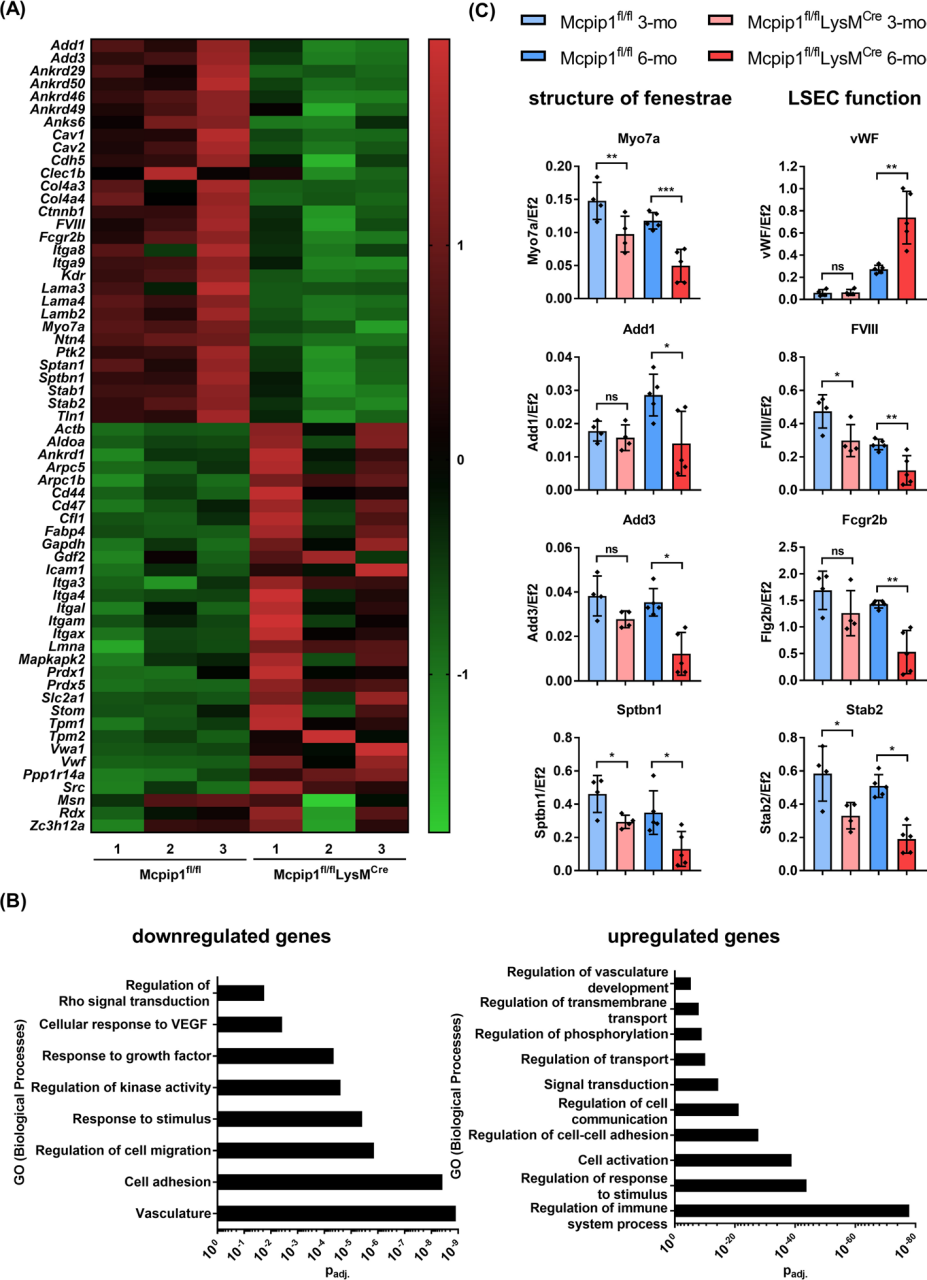
Hierarchical cluster analysis of integrated gene expression data from 6-month-old Mcpip1^fl/fl^ and Mcpip1^fl/fl^ LysM^Cre^. **A** Heat map showing down- and upregulation of manually selected genes reported to be involved in LSEC pathology and speculated to have a role in the regulation of LSEC porosity. **B** Up- and downregulated biological processes changed in LSECs from the Mcpip1 KO group. **C** Comparison of selected gene expression for 3-month-old and 6-month-old by RT-PCR. *n* = 4–5 animals

To find genetic hallmarks that determine impaired LSEC recovery after cytochalasin B treatment, we reanalysed NGS data from 6-month-old controls and Mcpip1 KO previously published by Zurawek and co-workers [29]. We selected several genes based on the literature data, which indicate their potential role in regulating fenestration (Fig. 6A, B). We compared their expression between LSECs isolated from 3-month-old and 6-month-old mice using RT-PCR (Fig. 6C).

Many of the downregulated genes in LSECs isolated from 6-month-old Mcpip1 KO mice have been previously reported to determine LSEC endocytosis and scavenger function (i.e. *FVIII, Fcgr2b, Stab1, Stab2*) (Fig. 6A). Moreover, we identified a repertoire of downregulated genes responsible for the cytoskeleton organisation, which may also play a role in the structure of fenestrae (i.e. *Myo7a*, *Add3*, *Add1*, *Sptbn1*) [2]. On the other hand, some of the genes involved in the cytoskeleton formation were upregulated (i.e. *Actb*, *Itga3*, *Itga4*, *Lmna*, *Tpm1* and *Tpm2*), indicating a significant rearrangement of the cytoskeleton, which could result in impaired fenestrae formation. Among upregulated genes, we also detected antioxidant genes (i.e. *Prdx1* and *Prdx5*) and adhesion molecules (i.e. *Cd44*, *Cd47* and *Icam1*).

Gene ontology analysis further confirmed that the most altered cellular processes in LSECs isolated from 6-month-old Mcpip1 KO were those that depend on the organisation and regulation of the cytoskeleton (Fig. 6B). Among the most significantly downregulated processes, we identified vasculature, cell adhesion and regulation of cell migration. However, significantly downregulated genes were also associated with kinase activity and response to growth factors, which are important signalling processes regulating LSEC fenestrations [64, 65]. On the other hand, upregulation of cell activation, cell-to-cell adhesion, transport, signal transduction and transmembrane trafficking was detected (Fig. 6B). Additional functional enrichment analysis based on differentially expressed genes (DEGs) confirmed that transport regulation is one of the functions significantly enriched in LSECs isolated from 6-month-old Mcpip1 KO (Supplementary Figs. 4 and 5).

Further comparative RT-PCR analysis of selected genes belonging to the LSEC cytoskeleton network demonstrated that myosin VIIa (*Myo7a*) and non-erythrocytic spectrin beta-1 (*Sptbn1*) were significantly reduced in LSECs isolated from both 3- and 6-month-old Mcpip1 KO mice compared with the respective controls (Fig. 6C). Adducin alpha (*Add1*) and adducin gamma *(Add3*) were significantly reduced in 6-month-old Mcpip1 KO, but not affected in 3-month-old Mcpip1 KO compared with the respective controls. From the genes described as regulating LSEC function, factor VIII (*FVIII*) was reduced in both time points, but the Fc-gamma receptor (*Fcgr2b*) was significantly reduced only in 6-month-old Mcpip1 KO. Von Willebrand factor (*vWF*) was significantly increased only in LSECs isolated from older Mcpip1 KO compared with age-matched controls. Interestingly, the expression of *Vwf* increased with age, whereas *FVIII* expression decreased in both controls and Mcpip1 KO mice.

## Discussion

In the present work using AFM, SEM and quantitative fluorescence microscopy of actin filaments, we comprehensively analysed the progression of LSEC defenestration in Mcpip1^fl/fl^ LysM^Cre^ (Mcpip1 KO) mice underscoring the distinct phenotype of the early and late phases of defenestrated LSECs. Our results suggest that the progression of LSEC defenestration may determine the limited effectiveness of pharmacological refenestration therapies, detected at the level of primary isolated LSECs. The impairment of LSEC potential to form fenestrations cannot be attributed to a single feature but rather to a whole panel of progressive changes. These include significant reorganization of the actin cytoskeleton, cell stiffening, and altered gene expression, particularly pronounced in the late phase of LSEC defenestration. Unlike the early phase, where defenestration is fully reversible with cytochalasin B treatment, these changes in the late phase are not fully reversible.

High levels of actin polymerisation and the presence of thick fibres have been correlated with a reduced number of fenestrations in LSECs [25, 66]. Therefore, for almost 40 years now, actin disruptors have been successfully used to induce new fenestrations. Among them, cytochalasins became a gold standard, resulting in cell flattening and increasing accessibility of numerous short actin filaments to be organised into so-called ‘fenestrae-associated cytoskeletal rings’ (FACRs), initiating fenestration formation [67]. Here, we showed that indeed the abundance of long actin fibres was significantly higher in the Mcpip1 KO groups in both time points, which might explain the gradually decreasing number of open fenestrations. Although the effect of cytochalasin B on elastic modulus reduction and actin depolymerisation was similar in the control and the Mcpip1 KO groups, the effect on LSEC fenestrations was distinct. At an early stage of the model progression, the effect of cytochalasin B was similar in Mcpip1 KO and control groups. It might suggest that the increased polymerisation of actin filaments and stiffening of cells precedes the effect on reduced porosity. However, changes in LSEC phenotype at the late stage of systemic inflammation are profound and involve critical mechanisms that cannot be reversed to the same level as in control cells. As a result, depolymerisation of actin by cytochalasin B and observed softening of LSECs allowed for only partial recovery of LSEC fenestration. Previous studies reported several mechanisms and actin-binding proteins involved in the formation of LSEC fenestrations, however, none of them have been identified as critical so far [1, 19–21, 66, 68]. In general, the attachment of actin to the cell membrane may be facilitated in several ways, involving spectrin, ankyrin and adducin, and has been well-studied in red blood cells [69–71]. In particular, spectrin plays an important role in bridging actin filaments when organizing FACRs in primary LSECs, and disruption of spectrin by diamide or iodoacetic acid was sufficient to affect fenestrations [66]. Here, we highlighted the downregulation of all spectrin genes, especially spectrin beta1 (*SPTB1*), in LSECs isolated from both young and old Mcpip1 KO mice. Thus, the reduced LSECs porosity might be related not only to the impaired protein function or structure but also to the limited availability of the proteins being building blocks for fenestrations. The pharmacological destabilisation of the actin cytoskeleton may have an indirect effect on the organisation of spectrin [70, 72]. In particular, Zhao et al. showed that staurosporine and latrunculin B but not cytochalasin B, disrupted actin filaments and dissolved the spectrin-like cytoskeleton over the cell membrane [70]. Our previously published fluorescence imaging of LSEC cytoskeleton confirmed that spectrin was not affected and remained uniformly distributed after cytochalasin B treatment, suggesting that the organisation of spectrin itself does not determine the reversibility of LSEC defenestration [66, 70].

At the late stage of systemic inflammation LSECs were characterised by the downregulation of ankyrin-related genes (*Ankrd29*, *Ankrd50*, *Ankrd46*, *Ankrd49* and *Anks6*), responsible for anchoring spectrin and actin to the cell membrane through a band 3 protein [73]. However, owing to the rather low overall expression of ankyrins in LSECs [29, 74], it seems unlikely that they can be a limiting factor in fenestration formation. Actin can also be bound to the cell membrane through the band 4.1 complex, which involves adducin and proteins of the so-called FERM domain (ezrin, radixin and moesin). Among FERM domain proteins, only radixin and moesin were identified in liver tissue [75]. Moesin expression was mostly restricted to LSECs, whereas radixin was present in bile canaliculi and in small amounts in LSECs [75]. Our data from RNAseq showed that the late stage of Mcpip1-induced systemic inflammation was associated with only mild changes in *Msn* and *Rdx* gene expression in LSECs. However, *Myo7a*, encoding myosin VII protein with two FERM domains, was downregulated in a time-dependent manner. Myosin VII is abundant in LSECs [74] and it was reported to colocalise with the phosphorylated myosin light-chain (MLC) at the edges of sieve plates [20]. Dephosphorylation of MLC was shown to be involved in the regulation of fenestrations diameter and number [20]. Lower expression of *Myo7a* might be connected with an impaired increase in the formation of new fenestrations, by which hamper the process of sieve plate formation.

The final potential candidate highlighted in this report is adducin. We observed downregulation of adducin alpha (*Add1*) and gamma (*Add3*) in 6-month-old Mcpip1 KO mice without being affected in the younger group. Adducin facilitates the assembly of actin and spectrin and the binding of this network to the cell membrane through FERM domains [76, 77]. It was demonstrated in keratinocytes that the KO of adducin affected both the expression of adducin and the organisation of spectrin [70]. Moreover, adducin is a substrate for protein kinase A and protein kinase C and its phosphorylation is controlled by Rho kinase and calcium-calmodulin binding, also involved in the regulation of fenestrations [1, 19, 20, 24]. Importantly, circulating cytokines, endotoxins and invading bacteria may induce degradation of adducin [78–80]. Thus the deterioration and irreversibility of LSEC defenestration observed in 6-month-old, but not 3-month-old Mcpip1 KO mice could be related to the potentiated inflammation resulting in the loss of adducin.

Alterations in LSEC morphology are strongly associated with functional changes, as capillarisation with defenestration has been observed in liver cirrhosis, steatosis and NASH [7, 81–84]. In particular, the report of Verhulst et al., from 2021 documented four genes (*Fabp4*, *Fabp5*, *Vwf* and *VwA1*) which should be considered as markers of LSEC damage [82]*.* In our study, we confirmed this pattern showing upregulation of *Fabp5*, *Vwf* and *VwA1* in LSEC from 6-month-old Mcpip1 KO mice. Moreover, high levels of factor VIII (*FVIII*) and low levels of von Willebrand factor (*VWF*) are characteristics of healthy LSECs [85], whereas in cirrhotic livers this ratio is reversed [86]. Observed in our study a gradual decrease in the level of *FVIII* and increased expression of *VWF* was in pair with the reduction in fenestration number and attenuated responsiveness to cytochalasin B. As *FVIII* is expressed in fenestrated cells in liver sinusoids and glomerular endothelial cells or lymphatic endothelial cells, it can be associated with fenestrations. High levels of *VWF* are present in endothelial cells in large vessels and upregulation of *VWF* in LSECs may be considered as a marker of capillarisation. However, a recent report showed that refenestration is possible in metabolic dysfunction-associated steatohepatitis (MASH), termed before as non-alcoholic steatohepatitis (NASH) [81] indicating that LSECs still have the potential to induce fenestrations despite impaired expression of *FVIII* and *VWF*. Additionally, we observed that expression of *Fcgr2b*, encoding the signature receptor for endocytosis, was reduced in LSECs isolated from older but not from young Mcpip1 KO. These results, together with the observed reduced expression of stabilin, may indicate the severe impairment of LSEC endocytosis. However, the recent study contradicted the existence of a correlation between *FcγRIIb* expression and LSEC porosity, as significant downregulation of *FcγRIIb* was not accompanied by LSEC defenestration in a mouse model of periportal liver fibrosis [87]. Moreover, the lack of visible changes in acLDL uptake in defenestrated LSECs isolated from mouse model of chronic heart failure [10] suggests that endocytic activity does not affect fenestration functionality.

Previously reported data indicated, that rat LSEC phenotype, including open fenestrae in sieve plates, is maintained by autocrine and paracrine regulation [88]. It was shown that hepatocytes or stellate cells produce vascular endothelial growth factor (VEGF), which stimulates LSECs to release of nitric oxide (NO), a downstream autocrine determinant of LSEC phenotype [89]. Gene Ontology data revealed the downregulation of genes associated with response to growth factors in Mcpip1 KO animals, thus we analysed changes in VEGF/NO signalling molecules. We observed a decrease in the expression of the *Kdr*, which encodes the VEGFR2 receptor. This may lead to an attenuated response of LSECs to the growth factor. What is more, lower β-catenin expression was also observed in Mcpip1 KO mice in comparison with controls, and Wnt/β-catenin signalling was recently shown to be important in endothelial cells for the regulation of permeabilisation [90]. Thus, despite elevated *Src* expression in Mcpip1 KO-derived LSECs, low β-catenin could abolish fenestrations. Additionally, bioinformatic analysis indicated higher *Cdh5* gene expression in Mcpip1 KO group, which encodes VE-cadherin protein – the most important adhesion molecule in the formation of vascular structure [91]. The implication of increased cell-to-cell adherence as well as the merging of neighbouring fenestrae and forming gaps in LSECs was shown before in partial hepatectomy [92]. Altogether, in light of the provided discussion, it seems unlikely that disruption of individual LSEC functions would affect LSEC porosity. Therefore, rather impaired cytoskeletal regulatory mechanisms or downregulation of structural proteins are responsible for attenuated LSEC response to cytochalasin B treatment in the 6-month-old (but not in the 3-month-old) Mcpip1 KO group.

Apart from the identification of potential components of the fenestration structure, we applied a novel AFM-based imaging technique to test the ‘forced sieving’ hypothesis in vitro. Both technical and physiological aspects, such as the endocytic activity of LSECs, the difficulty in forming monolayers and the rapid loss of phenotype by primary LSECs, or the inability to track the fate of individual particles in the hepatic microcirculation, have made the hypothesis of ‘forced sieving’ not addressed until now. There is growing research to fabricate artificial sinusoids to mimic liver microcirculation [93, 94], but under static or even fluid flow conditions, one lacks the aspect of force exerted by moving blood particles. Therefore we proposed the experiment on the basis of AFM, which enables to exert localised perpendicular force on each position of the cell surface and to reconstruct images for increasing load force. Our observations suggest that the stiffening of whole LSECs is translated to the stiffness of individual fenestrations. Therefore the transfer of lipoproteins through fenestrations might be hampered not only by altered fenestration size and number but also by impaired mechanoresponsive elements regulating dynamics of LSEC fenestration in vivo.

## Conclusions

Our study demonstrated that LSECs derived from Mcpip1 KO mice are characterised by two phases of defenestration: early, with modest defenestration that is fully reversible using cytochalasin B and late, with severe defenestration that is irreversible. Our findings showed that the functional response of LSECs to cytochalasin B is determined not by a single feature but by a number of changes involving not only the structure of the actin cytoskeleton but also its regulation at the gene level. Importantly, attenuated responsiveness to the actin-targeted treatment with accompanied phenotypic changes could be demonstrated at the level of primary isolated LSECs, opening the novel avenue for in vitro pharmacological studies on refenestration strategies using this murine model of robust systemic inflammation encompassing liver pathology. In addition, here, we presented to the best of our knowledge, for the first time results suggesting the importance of altered mechanoresponsive elements in the pathophysiology of hampered forced sieving properties of defenestrated LSECs, which constitute a key function of LSECs and may be studied by AFM-based approach. Moreover, these results allowed us to link the deformability of fenestrations with the overall stiffness of LSECs in the liver. Taking into account the physiological consequences of LSEC defenestration leading to impaired lipid homeostasis, maintaining proper LSEC porosity and its restoration in the course of various diseases seems to be important. Therefore, studies using models of gradual changes in LSEC porosity, demonstrating distinct effects of the drugs on fenestrations, should be used to identify the structural components of functional fenestrations and to develop novel strategies for refenestration in liver pathologies.

## Supplementary Information

Additional file 1.

## Data Availability

The datasets used and/or analysed during the current study are available from the corresponding author on reasonable request.

## Abbreviations

AFM: Atomic force microscopy
BP: Biological process
DEGs: Differentially expressed genes
FACR: Fenestrae-associated cytoskeletal rings
FERM: F for 4.1 protein, E for ezrin, R for radixin and M for moesin
GA: Glutaraldehyde
GO: Gene Ontology
HEPES: 4-(2-Hydroxyethyl)piperazine-1-ethane-sulfonic acid
KO: Knock-out
LSEC: Liver sinusoidal endothelial cell
LysM: Lysozyme M, a cell-specific marker for myeloid cells
(MASH): Metabolic dysfunction-associated steatohepatitis
Mcpip1: Monocyte chemoattractant protein-1 induced protein 1
MLC: Myosin light-chain
NASH: Non-alcoholic steatohepatitis
NO: Nitric oxide
PLVAP: Plasmalemma vesicle-associated protein
ROI: Region of interest
RT-qPCR: Reverse transcription-quantitative polymerase chain reaction
SEM: Scanning electron microscopy
QI: Quantitative imaging
VEGF: Vascular endothelial growth factor
*VWF*: Von Willebrand factor
