# Supplementary material for: Early and late phases of liver sinusoidal endothelial cell (LSEC) defenestration in mouse model of systemic inflammation
Source: Cell Mol Biol Lett. 2024 Nov 11;29:139. doi: 10.1186/s11658-024-00655-w (PMC11556108; doi:10.1186/s11658-024-00655-w)
Supplement: Supplementary file 1 — Additional file 1. [file 11658_2024_655_MOESM1_ESM.zip › Supplementary figure 1.pptx]

## Slide 1
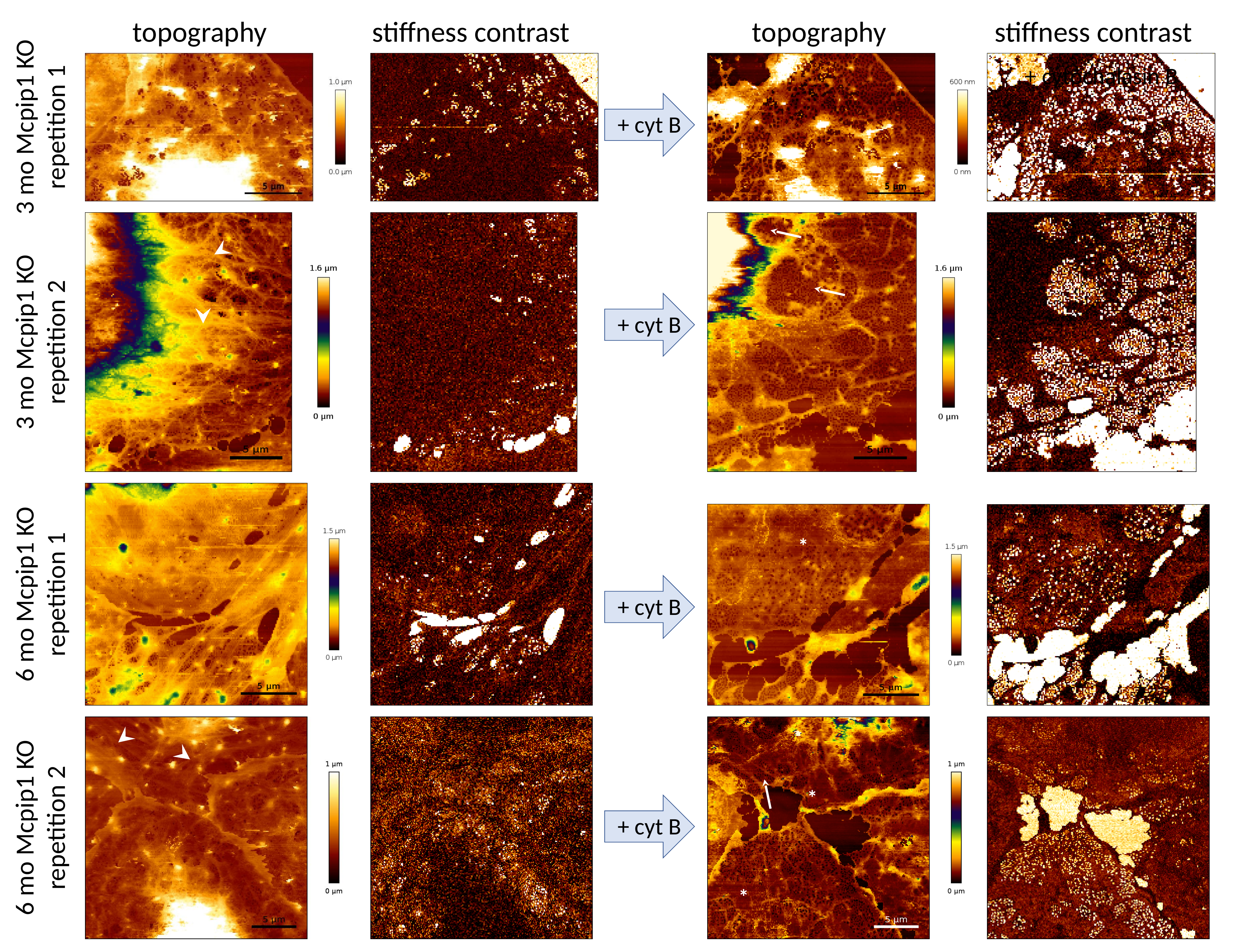

topography
stiffness contrast
topography
stiffness contrast
+ cytochalasin B
3 mo Mcpip1 KO
repetition 1
+ cyt B
+ cyt B
3 mo Mcpip1 KO
repetition 2
*
6 mo Mcpip1 KO
repetition 1
+ cyt B
*
6 mo Mcpip1 KO
repetition 2
+ cyt B
*
