# Supplementary material for: Early and late phases of liver sinusoidal endothelial cell (LSEC) defenestration in mouse model of systemic inflammation
Source: Cell Mol Biol Lett. 2024 Nov 11;29:139. doi: 10.1186/s11658-024-00655-w (PMC11556108; doi:10.1186/s11658-024-00655-w)
Supplement: Supplementary file 1 — Additional file 1. [file 11658_2024_655_MOESM1_ESM.zip › Supplementary information_rev2.docx]

**Supplementary information**

**Early and late phases of liver sinusoidal endothelial cell (LSEC) defenestration in mouse model of systemic inflammation**

Izabela Czyzynska-Cichon^§1^, Jerzy Kotlinowski^§2^, Oliwia Blacharczyk^3^, Magdalena Giergiel^4^, Konrad Szymanowski^3^, Sara Metwally^3^, Kamila Wojnar-Lason^1^, Ewelina Dobosz^5^, Joanna Koziel^5^, Malgorzata Lekka^3^, Stefan Chlopicki^1^, Bartlomiej Zapotoczny*^3^

^1^ Jagiellonian Centre for Experimental Therapeutics (JCET), Jagiellonian University, Bobrzynskiego 14, 30-348 Krakow, Poland

^2^ Department of General Biochemistry, Faculty of Biochemistry, Biophysics and Biotechnology, Jagiellonian University, Gronostajowa 7, 30-387 Krakow, Poland

^3^ Institute of Nuclear Physics Polish Academy of Sciences, PL-31342 Krakow, Poland

^4^ Centre for Nanometer-Scale Science and Advanced Materials (NANOSAM), Faculty of Physics, Astronomy, and Applied Computer Science, Jagiellonian University, Krakow, Poland

^5^ Department of Microbiology, Faculty of Biochemistry, Biophysics and Biotechnology, Jagiellonian University, Gronostajowa 7, 30-387 Krakow, Poland

^§^ These authors contributed equally

* Corresponding author: Bartlomiej Zapotoczny, Ph.D.,
email: bartlomiej.zapotoczny@ifj.edu.pl, tel. +48 12 662 81 96

Table of contents

[1. Materials and Methods 2](#_Toc168486389)

[1.1 Atomic Force Microscopy 2](#_Toc168486390)

[1.2 Reverse Transcription-quantitative Polymerase Chain Reaction (RT-qPCR) 4](#_Toc168486391)

[2. Supplementary figures 5](#_Toc168486392)

[***Supplementary figure 1*** 5](#_Toc168486393)

[***Supplementary figure 2*** 6](#_Toc168486394)

[***Supplementary figure 3*** 7](#_Toc168486395)

[***Supplementary figure 4*** 8](#_Toc168486396)

[***Supplementary figure 5*** 9](#_Toc168486397)

[3. Supplementary videos 10](#_Toc168486398)

[***Supplementary video 1*** 10](#_Toc168486399)

[***Supplementary video 2*** 10](#_Toc168486400)

[***Supplementary video 3*** 10](#_Toc168486401)

[***Supplementary video 4*** 10](#_Toc168486402)

## 1. Materials and Methods

### 1.1 Atomic Force Microscopy

**A) Quantitative imaging (QI) mode imaging of fixed cells**

The culture medium (EGM-2, Lonza) was replaced with fresh medium with or without the cytochalasin B and fixed after 30 minutes using 2% glutaraldehyde before measurements. SCM-PIC-V2 (Bruker) cantilevers (k = 0.1 N/m, nominal tip apex radius of 25 nm) were used for the imaging, according to the methodology described before (Zapotoczny et al., 2019). Briefly, each image was acquired by performing multiple force curves in each pixel(px)/point of the image that were translated into the images of topography and stiffness. Load force was adjusted for individual cantilevers to achieve the best spatial resolution without distortion of fenestrations and was in the range of 200-350 pN. The length of the force curves (the *z* range) and the acquisition speed were in the range of 950-1050 nm and 120-140 µm/s, respectively. Image acquisition lasts 35-65 minutes depending on the scan frame size and pixel density. As established before, we measured large areas covering at least one LSEC, not smaller than 1000 µm^2^ (Zapotoczny et al., 2017a). Measurements were conducted at 25°C, set using PetriDish Heater™ (JPK Instruments). Images were analysed using an automatic method based on a neural network, as described before (Giergiel et al., 2021).

**B) QI mode imaging of living cells**. The culture medium was replaced with the fresh one (EGM-2c, Lonza) with the addition of 25 mM of HEPES buffer (4-(2-Hydroxyethyl)piperazine-1-ethane-sulfonic acid, Sigma-Aldrich) to prevent changes in pH of culture medium during the measurements (ambient atmosphere). The temperature was set to 37°C using PetriDish Heater™. Measurements were performed similarly to fixed cells. To increase the speed of image acquisition, both scan size and pixel density (resolution) were reduced. The selected area was scanned several times to track the alterations in cell morphology and position and number of fenestrations. To test the responsiveness of LSECs to drug treatment, the compound was injected into the cell culture and imaging of the same area was continued. The set of images was presented in the form of supplementary animations. To determine the deformability of fenestrations, high magnification images of individual sieve plates were performed in LSECs from control and Mcpip^fl/fl^LysM^Cre^ mice using the same cantilever for both groups. Each biological repetition included a selection of a new cantilever and a reversed sequence between the control and knockout group to avoid alterations caused by the modified tip geometry. The load force applied was constant and equal to 350 pN. The images were reconstructed for load forces of 170 nN and 300 pN, which represent ~50% and ~80% of the maximum load force respectively. A load force of 170 pN was selected as a minimal value allowing for the reduction of noise during the measurements and measurement stability for used cantilevers. The manufacturer recommends 80% of the maximal indentation to ensure stable reconstruction of QI images. Larger indentations were not considered, as they resulted in the merging of neighbouring fenestrations (Supplementary information in (Zapotoczny et al., 2017b)).

**C) Force spectroscopy**. The force spectroscopy mode of AFM allows for the determination of elastic modulus of investigated objects. Force-distance curves were acquired in the central area of the cell with sharp and hemispherical probes in force-volume mode. Sharp silicon nitride probes (MLCT-BIO-DC-C, k = 0.01 N/m, Bruker) were used with a load force of 1.0 nN (resulting in the indentation of 1000-1500 nm) to investigate the elastic modulus. The elastic modulus can be considered apparent Young’s modulus, as not all requirements of the contact mechanics can be fulfilled for biological objects (Rusaczonek et al., 2019b). Additionally, part of the experiments was repeated using novel, pre-calibrated, hemispherical silicon nitride probes, SAA-SPH-5UM (Bruker), with nominal spring constant k = 0.25 N/m, tip radius of 5.0 µm, and tip height of 25 µm. Measurements using hemispherical probes were performed with a load force of 2.0 nN (resulting in the indentation of 400-600 nm) to focus on the elasticity of the cortical layer of the cells (Pesen and Hoh, 2005; Rusaczonek et al., 2019a). 5×5 µm^2^ matrix in the central area of cells was selected and 25 force-distance curves were acquired per cell with a *z* length of 5.0 µm and acquisition speed of 8.0 µm/s. Elastic modulus was calculated according to the Hertz-Sneddon model of contact mechanics using *JPK Processing Software*. Before measurements, the calibration force-distance curve was acquired on a glass (non-deformable) surface for the MLCT-BIO-DC-C cantilevers. The spring constant was calibrated using the thermal tune (Schillers et al., 2017). The hemispherical probes were pre-calibrated by the manufacturer. Then, the subtraction of the calibration curve from curves collected on living cells produces the relation between load forces and indentations depths. Elastic modulus was calculated according to the Hertz-Sneddon model of contact mechanics (Sneddon, 1965) using *JPK Processing Software*. The relation between load force and indentation depth is the following (the shape of the pyramidal probes was approximated by a cone):

**Sharp, conical probe:**

$$F\left( \delta\right)= \frac{2 \cdot\tan\alpha}{\pi} \cdot\frac{E_{cell}}{1- \mu^{2}} \cdot\delta^{2}$$

**Hemispherical probe**

$$F\left( \delta\right)= \frac{4}{3} \cdot\frac{E_{cell}}{1- \mu^{2}} \cdot\sqrt{R} \cdot\delta^{\frac{3}{2}}$$

where *F* is the load force, *δ* is the indentation depth, *E_cell_* is the elastic modulus of the cell, and *µ* is the Poisson's ratio (assumed to equal 0.5 for incompressible materials). Moreover, for conical probes, α is the tip half angle and R is the tip radius of curvature for the hemispherical probes.

### 1.2 Reverse Transcription-quantitative Polymerase Chain Reaction (RT-qPCR)

Primer sequences (Genomed/Sigma) used for qPCR are listed in Supplementary Table 1 below.

***Supplementary table 1.*** Sequences of primers used for qPCR.

| **Gene** | **Forward (5’->3’)** | **Reverse (5’->3’)** |
| --- | --- | --- |
| *Ef2* | GACATCACCAAGGGTGTGCAG | TCAGCACACTGGCATAGAGGC |
| *Add1* | ATGACTATCATGGCATTCTG | GAATGAGGACCTTGCTTTTAG |
| *Add3* | GTACATCTCGGTACGAATAAG | CTGTCGCTTCAGAAAAAGAG |
| *Stab2* | CGTTTTGAGTCAGAAGATGAC | GTTGAGGTCTCATACAAAGG |
| *Fcgr2b* | CAAAACTGAGGCTGAGAATAC | AATATCTACAGCATCCCTTGG |
| *FVIII* | CCAGCATGTTTGTGAAAGAGTTCC | GAGTCCTGATTCCCCTGAAAAAC |
| *Sptbn1* | CTGAGCAGGGATCTCCACGG | TTGGCCTTTCGGTCCAAGGT |
| *Myo7a* | GATCTCCAACTGGAGTAGTG | GATCATCCATTTTGTATCCCAG |
| *vWF* | CTCCAGCCACATTCCATACC | GAGATGGGCGTAAGAAGCAA |

## 2. Supplementary figures

***
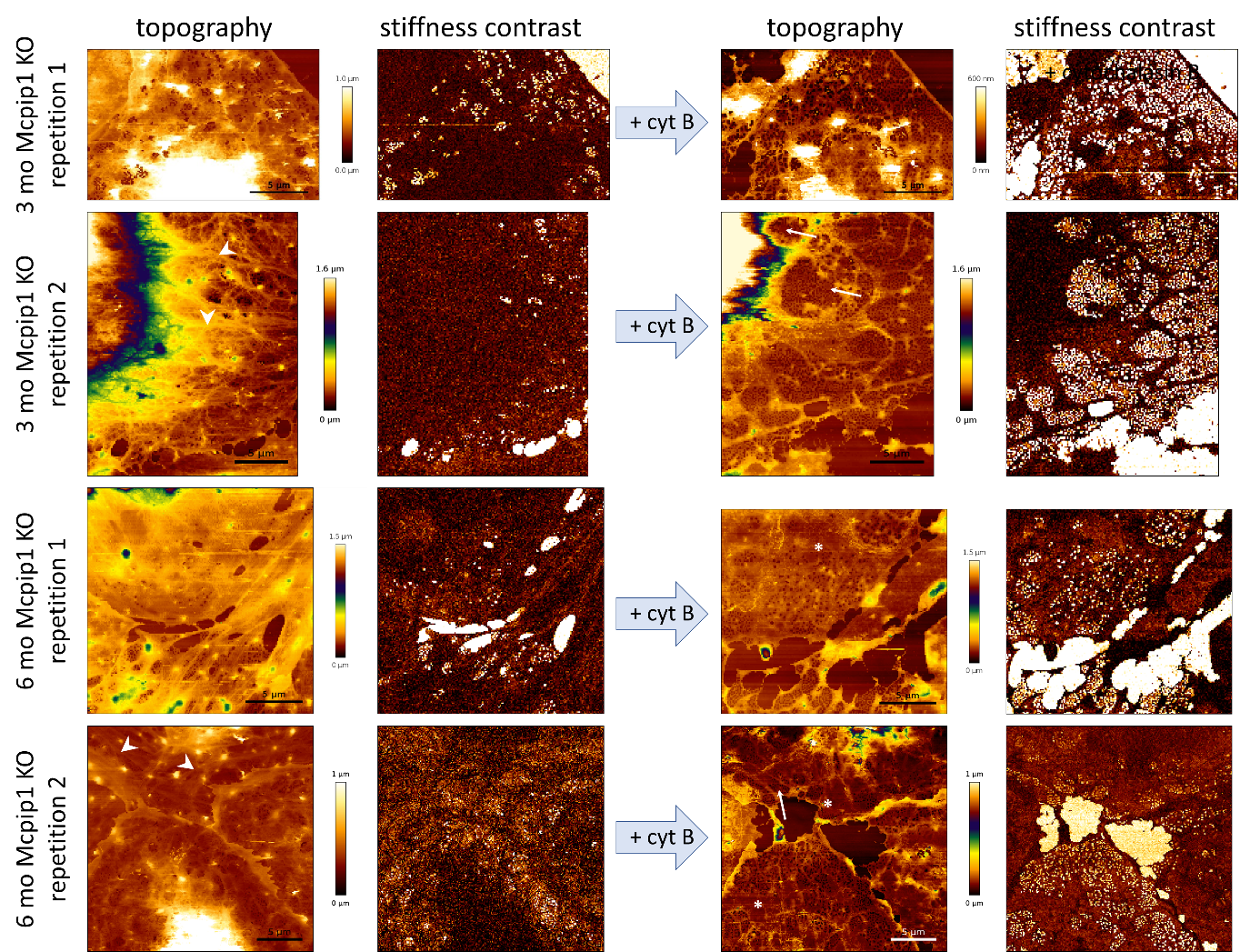
***

***Supplementary figure 1*** *AFM images selected from Supplementary videos 1-4. The images present topography and stiffness contrast of part of LSEC before and after the effect of 21 µM cytochalasin B. Topography allows distinguishing high areas of the cell including stress fibres and fenestrations. The stiffness channel (linear fit to the 10 nm of maximal indentation) creates a great contrast between stiff glass slide (bright) and soft cell (dark brown) allowing for easy identification of fenestrations.* *Cytochalasin B induced rapid changes in cellular morphology with the depolymerisation of thick fibres observed within the first 10 minutes* *(****Supplementary figure 1, arrowheads****). The effect of cytochalasin B on a number of fenestrations reached the maximum after 15-30 minutes as reported previously (Braet et al., 1996b; Zapotoczny et al., 2017b). Exposed to Cytochalasin B, LSECs isolated from 3-mo Mcpip1 KO mice formed numerous fenestrations and fenestrae-forming centres (Braet et al., 1999) (****Supplementary figure 1, arrows****). The number of newly formed fenestrations was much smaller in the 6-mo Mcpip1 KO group (****Supplementary figure 1, Supplementary videos 3,4****) when compared to the 3-mo Mcpip1 KO group. Even an incubation time longer than 30 minutes with Cytochalasin B did not result in an increase in the number of fenestrations. The cell height outside the nuclear region was reduced and shapes resembling sieve plates were identified (****Supplementary figure 1, asterisk****), but the membrane within was often sealed without opening new fenestrations.*


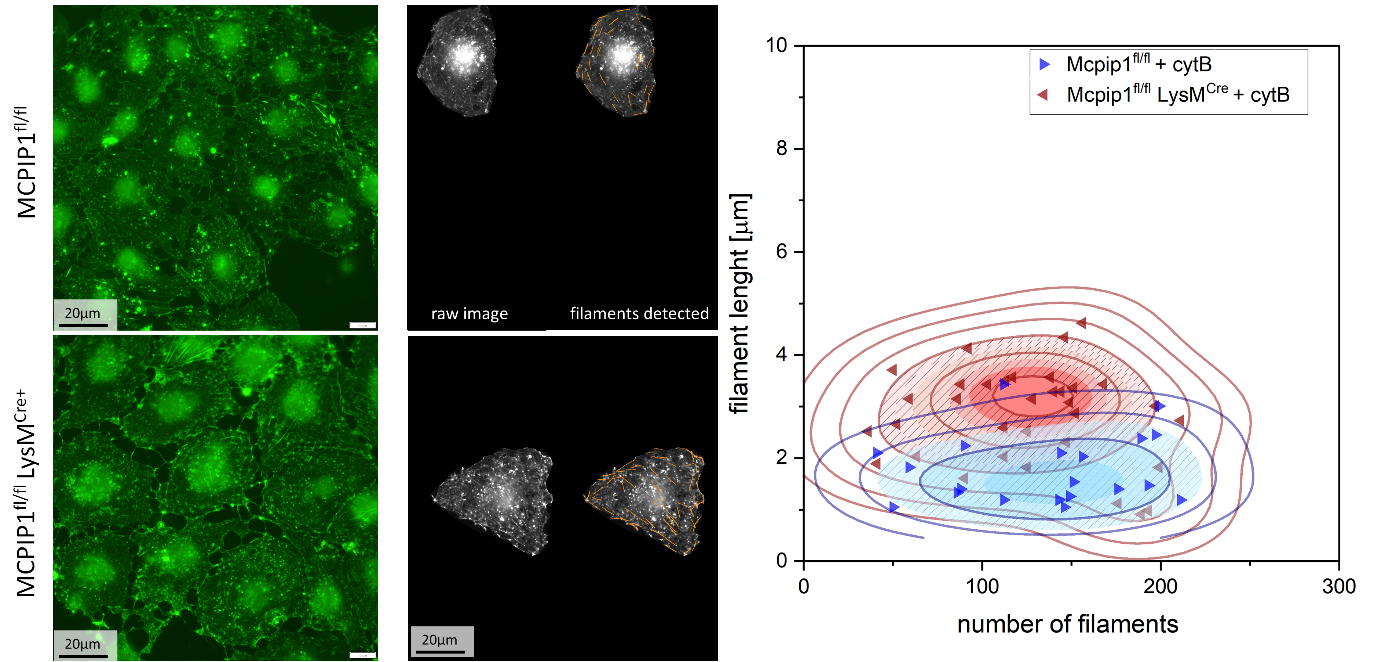


***Supplementary figure 2*** *Quantitative fluorescence of actin filaments for Mcpip1^fl/fl^ + cytB (top) and Mcpip1^fl/fl^ LysM^Cre^ + cyB (bottom). The panel presents representative images of cytochalasin B-treated LSECs from 6-month-old Mcpip1^fl/fl^ and Mcpip1^fl/fl^ LysM^Cre^ mice. A representative cell extracted from the fluorescence image was analysed using FilamentSensor software. The results of filaments length and number of 20 cells per group are presented as a Kernel density diagram (blue – Mcpip1^fl/fl^ +cytB, red – Mcpip1^fl/fl^ LysM^Cre^+cytB). Note that the y-scale range was 5 times smaller than presented in Figure 4 in the main document.*


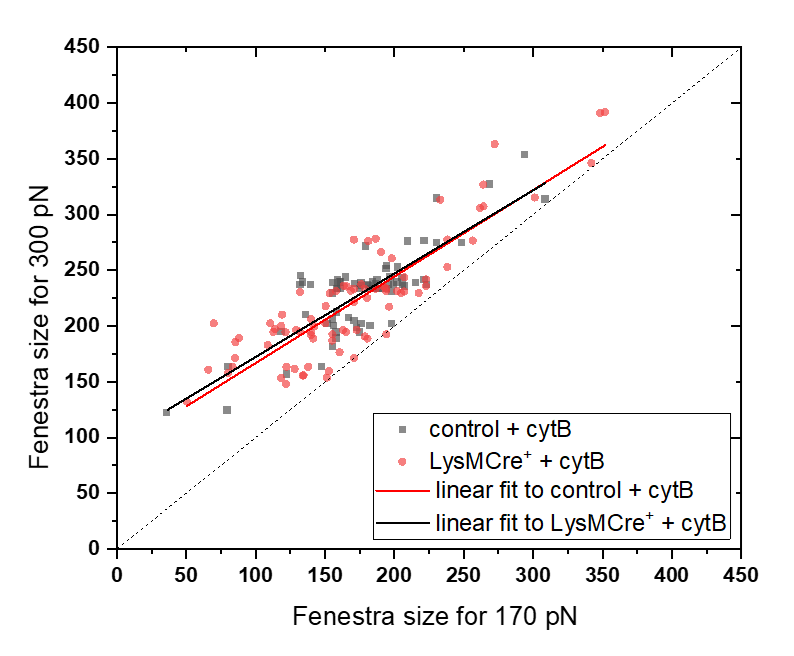


***Supplementary figure 3*** *Deformability of LSECs from Mcpip1^fl/fl^ LysM^Cre^ mice treated with cytochalasin B shows similar forced sieving, not dependent on the loading force used in the experiment as compared in a one-to-one manner.*


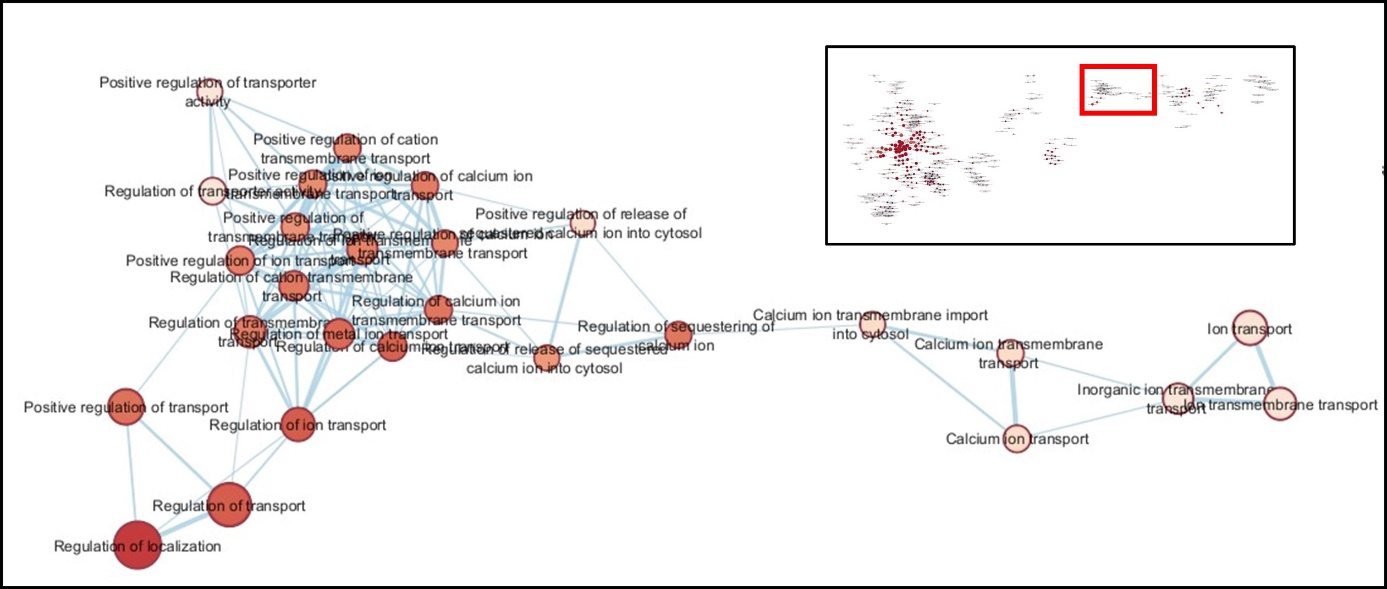


***Supplementary figure 4*** Functional enrichment analysis indicated that regulation of transport is one of the functions significantly enriched in a set of DEGs. **Supplementary figure 6** shows all pathways that were affected and Supplementary figure 4 shows a magnification of pathways involved in the regulation of cell transport. Because of the readability, **Supplementary figure 6** was provided as a separate file.

## 3. Supplementary videos

### ***Supplementary video 1***

A sequence of AFM images showing the effect of cytochalasin B on the morphology of LSECs derived from 3-month-old Mcpip1^fl/fl^ LysM^Cre^ mice. Images on the left show changes in the topography for the loading force of 208 pN, and images on the right show changes in the contrast of stiffness allowing easy distinction between glass slide (stiff, bright) and cell (soft, dark). After initial scanning and selecting the region of interest cytochalasin B was injected into the medium and imaging was continued. The depolymerisation of cytoskeleton fibres is observed and the formation of new sieve plates filled with fenestrations. Image size and resolution: 40.0 × 60.0 μm^2^, 180 × 270 points for large area (18 minutes per frame), 20.0 × 25.07 μm^2^, 150 × 188 points for selected area (approx. 8 minutes per frame). Total time: 110 minutes. Maximum loading force: 220 pN.

### ***Supplementary video 2***

A sequence of AFM images showing the effect of cytochalasin B on the morphology of LSECs derived from 3-month-old Mcpip1^fl/fl^ LysM^Cre^ mice. . Images on the left show changes in the topography for the loading force of 175 pN, and images on the right show changes in the contrast of stiffness allowing easy distinction between glass slide (stiff, bright) and cell (soft, dark). After initial scanning and selecting the region of interest cytochalasin B was injected into the medium and imaging was continued. The depolymerisation of cytoskeleton fibres is observed and the formation of new sieve plates filled with fenestrations. Cells became flat with bulging nuclei. Image size and resolution: 35.0 × 50.0 μm^2^, 128 × 183 points for large area (10 minutes per frame), 20.0 × 13.0 μm^2^, 180 × 117 points for selected area (approx. 8 minutes per frame). Total time: 102 minutes. Maximum loading force: 260 pN.

### ***Supplementary video 3***

A sequence of AFM images showing the effect of cytochalasin B on the morphology of LSECs derived from 6-month-old Mcpip1^fl/fl^ LysM^Cre^ mice. Firstly large area containing four cells was selected. Then the interconnection between cells was selected to focus on areas where fenestrations can be formed. Finally, we selected a single sieve plate with a few fenestrations to observe the effect of the drug. Then cytochalasin B was injected into the medium and imaging was continued. The depolymerisation of cytoskeleton fibres was observed and the formation of new fenestrations occurred. However, scanning again larger areas shows that the effect was distinct on different LSECs. Two cells had fenestrated areas on the periphery. One had no fenestrations. LSECs were flat with bulging nuclei. All cells have flat areas with sieve plates devoid of fenestrations. Image size and resolution: 40.0 × 40.0 μm^2^, 128 × 128 points for large area (6 minutes per frame), 20.0 × 20.0 μm^2^, 180 × 180 points for medium area (approx. 9 minutes per frame), 3.5 × 4.55 μm^2^, 90 × 117 points for selected area (approx. 3 minutes per frame). Total time: 101 minutes. Maximum loading force: 300 pN.

### ***Supplementary video 4***

A sequence of AFM images showing the effect of cytochalasin B on the morphology of LSECs derived from 6-month-old Mcpip1^fl/fl^ LysM^Cre^ mice. Firstly large area containing four cells was selected. Then the interconnection between cells was selected to focus on areas where fenestrations can be formed. Finally, we selected a single sieve plate with a few fenestrations to observe the effect of the drug. Then cytochalasin B was injected into the medium and imaging was continued. The depolymerisation of cytoskeleton fibres was observed but the formation of new fenestrations was limited. Instead, we observed a flat area. Scanning again larger areas shows that the effect is distinct on different LSECs, but all cells have flat areas with sieve plates devoid of fenestrations. Total time: 106 minutes. Maximum loading force: 206 pN. Image size and resolution: 60.0 × 60.0 μm^2^, 128 × 128 points for large area (7 minutes per frame), 25.0 × 25.0 μm^2^, 220 × 220 points for medium area (approx. 15 minutes per frame), 6.0 × 7.0 μm^2^, 100 × 116 points for selected area (approx. 4 minutes per frame). Total time: 106 minutes. Maximum loading force: 186 pN.
