# Supplementary figures and images for: Early and late phases of liver sinusoidal endothelial cell (LSEC) defenestration in mouse model of systemic inflammation
Source: Cell Mol Biol Lett. 2024 Nov 11;29:139. doi: 10.1186/s11658-024-00655-w (PMC11556108; doi:10.1186/s11658-024-00655-w)

## Slide 1
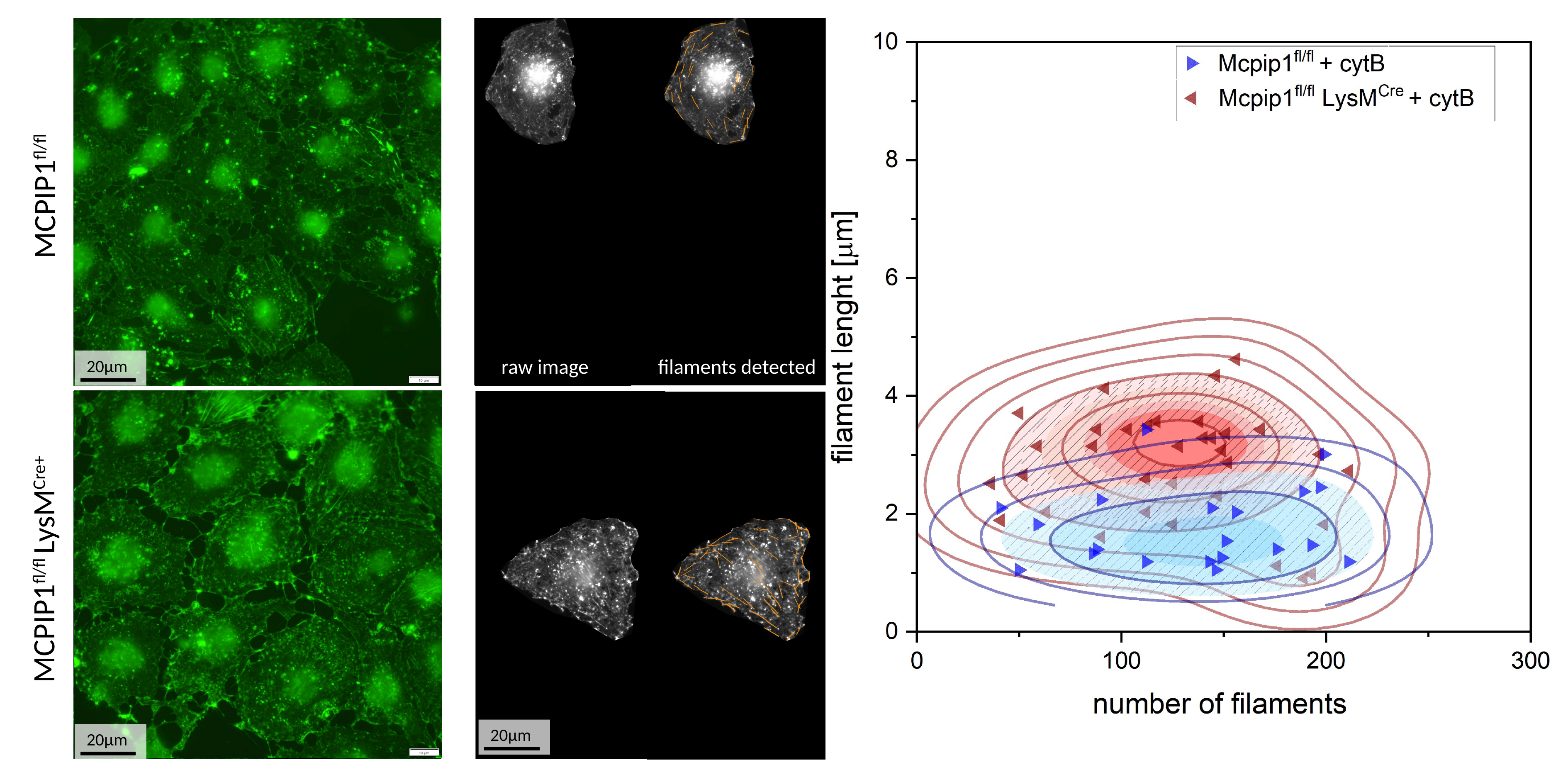

MCPIP1fl/fl
20µm
raw image
filaments detected
20µm
MCPIP1fl/fl LysMCre+
20µm
20µm

Supplement: Supplementary file 1 — Additional file 1. [file 11658_2024_655_MOESM1_ESM.zip › Supplementary figure 2.pptx]

## Slide 1
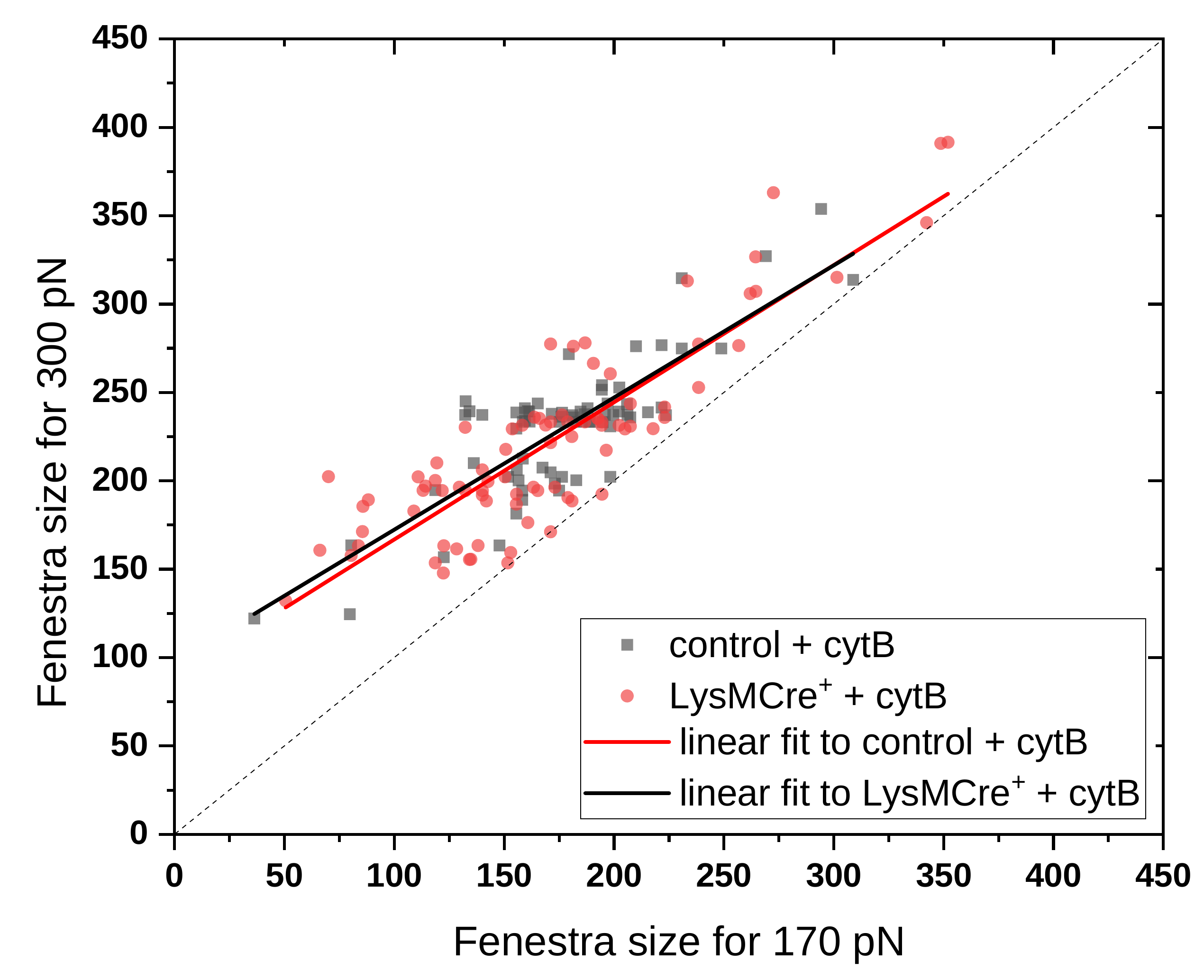

Supplement: Supplementary file 1 — Additional file 1. [file 11658_2024_655_MOESM1_ESM.zip › Supplementary figure 3.pptx]

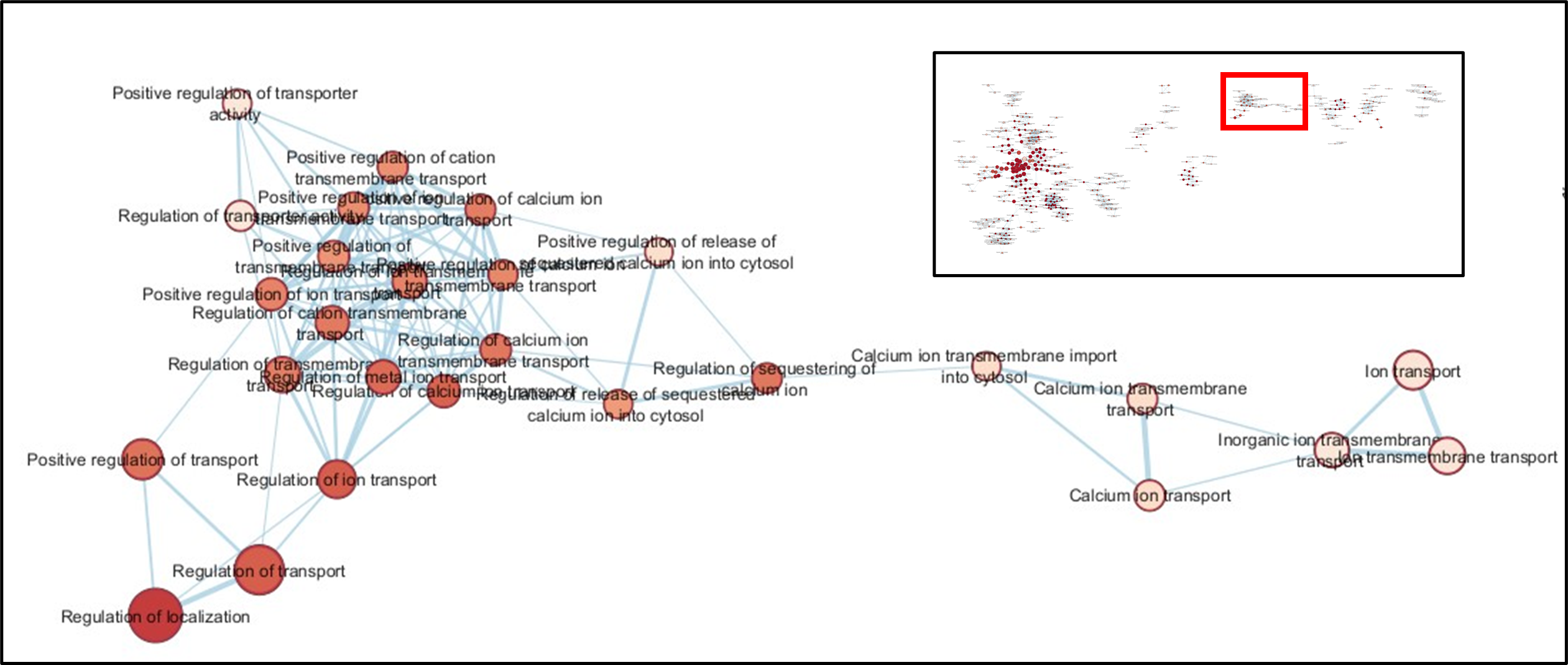

Supplement: Supplementary file 1 — Additional file 1. [file 11658_2024_655_MOESM1_ESM.zip › Supplementary figure 4.tif]

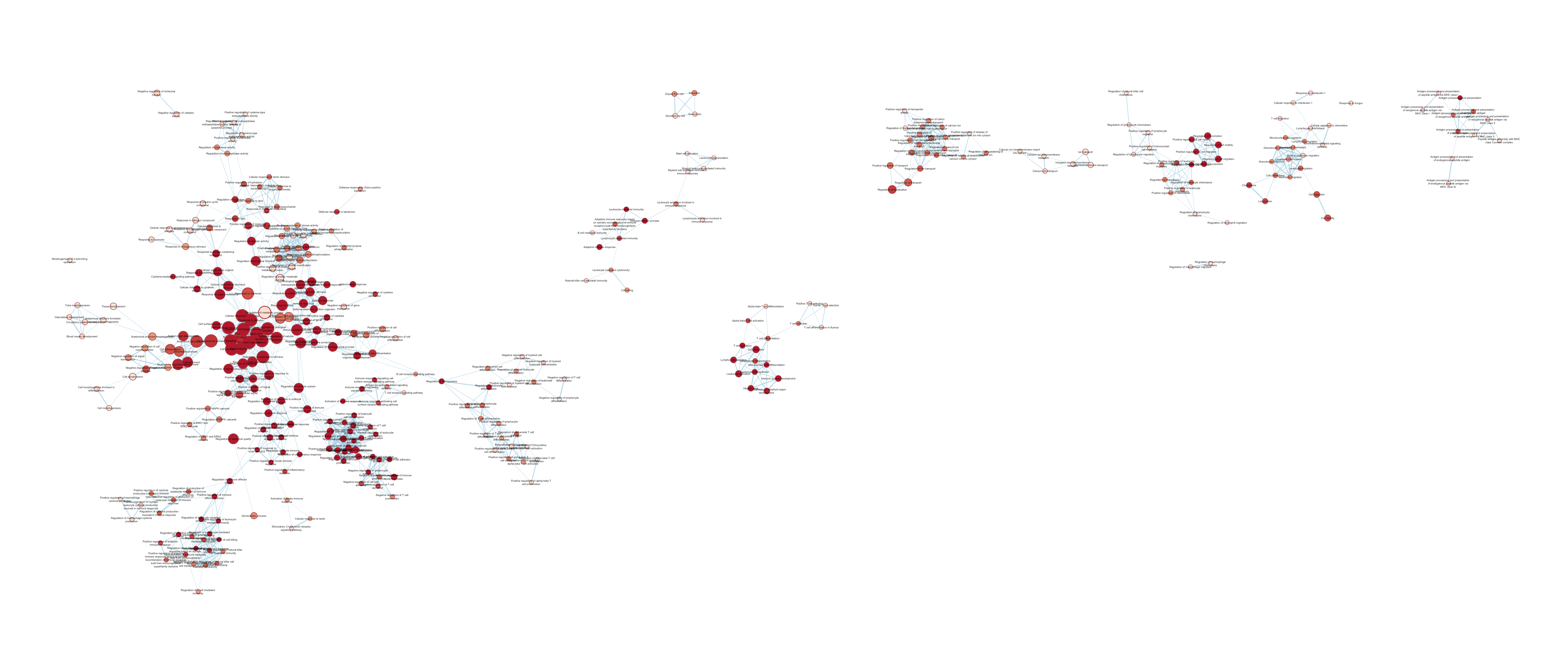

Supplement: Supplementary file 1 — Additional file 1. [file 11658_2024_655_MOESM1_ESM.zip › Supplementary figure 5.png]
